# Corporate Political Activity: Taxonomies and Model of Corporate Influence on Public Policy

**DOI:** 10.34172/ijhpm.2023.7292

**Published:** 2023-06-06

**Authors:** Selda Ulucanlar, Kathrin Lauber, Alice Fabbri, Ben Hawkins, Melissa Mialon, Linda Hancock, Viroj Tangcharoensathien, Anna B. Gilmore

**Affiliations:** ^1^Tobacco Control Research Group (TCRG), Department for Health, University of Bath, Bath, UK; ^2^School of Social and Political Science, University of Edinburgh, Edinburgh, UK; ^3^MRC Epidemiology Unit, University of Cambridge, Cambridge, UK; ^4^Trinity Business School, Trinity College Dublin, Dublin, Ireland; ^5^Alfred Deakin Institute, Deakin University, Melbourne, VIC, Australia; ^6^International Health Policy Programme, Ministry of Public Health, Nonthaburi, Thailand

**Keywords:** Commercial Determinants of Health, Public Health Policy, Tobacco, Alcohol, Ultra-Processed Foods, Gambling

## Abstract

**Background:** Non-communicable diseases (NCDs) kill 41 million people a year. The products and services of unhealthy commodity industries (UCIs) such as tobacco, alcohol, ultra-processed foods and beverages and gambling are responsible for much of this health burden. While effective public health policies are available to address this, UCIs have consistently sought to stop governments and global organisations adopting such policies through what is known as corporate political activity (CPA). We aimed to contribute to the study of CPA and development of effective counter-measures by formulating a model and evidence-informed taxonomies of UCI political activity.

**Methods:** We used five complementary methods: critical interpretive synthesis of the conceptual CPA literature; brief interviews; expert co-author knowledge; stakeholder workshops; testing against the literature.

**Results:** We found 11 original conceptualisations of CPA; four had been used by other researchers and reported in 24 additional review papers. Combining an interpretive synthesis of all these papers and feedback from users, we developed two taxonomies – one on framing strategies and one on action strategies. The former identified three frames (policy actors, problem, and solutions) and the latter six strategies (access and influence policy-making, use the law, manufacture support for industry, shape evidence to manufacture doubt, displace, and usurp public health, manage reputations to industry’s advantage). We also offer an analysis of the strengths and weaknesses of UCI strategies and a model that situates industry CPA in the wider social, political, and economic context.

**Conclusion:** Our work confirms the similarity of CPA across UCIs and demonstrates its extensive and multi-faceted nature, the disproportionate power of corporations in policy spaces and the unacceptable conflicts of interest that characterise their engagement with policy-making. We suggest that industry CPA is recognised as a corruption of democracy, not an element of participatory democracy. Our taxonomies and model provide a starting point for developing effective solutions.

## Background

**Key Messages**

**Implications for policy makers**

- The corporate political activity (CPA) model and taxonomies we present provide an evidence-informed and accessible tool to understand, document, predict, and counter policy influence strategies used by unhealthy commodity industries (UCIs).
- These tools will help policy-makers evaluate the reasons behind corporate stakeholders’ offers to engage with and support the policy-making process and to be alert to conflicts of interest in the policy space.
- The tools will encourage policy-makers to question and examine the validity of corporate data, critiques, statements, and legal threats.
- The tools will also enable policy-makers to identify presence of ‘corporate fingerprints’ on apparently independent statements and evidence that oppose policies.
- Finally, policy-makers can use these tools to successfully promote and safeguard public health policies.

**Implications for the public**

Many non-communicable diseases (NCDs) including mental health problems are caused by tobacco, ultra-processed foods and beverages alcohol and gambling. NCDs kill 41 million people every year. Governments can use policies to reduce use of these products and prevent illness, but the manufacturing corporations repeatedly block, weaken, and delay such policies through what is known as corporate political activity (CPA). We reviewed scholarly articles and interviewed experts and found that these four industries used similar influencing strategies. We developed taxonomies and a comprehensive model of CPA which show, inter alia, that they disseminate inaccurate statements and data about the health harms of their products and the effectiveness of policies, often hiding behind apparently independent public organisations or individuals, and try to shape policies by engaging with policy-makers or threatening to sue them. Our taxonomies and model can help advocates and policy-makers understand and counteract CPA and successfully introduce policies that protect the public’s health.

Non-communicable diseases (NCDs) kill 41 million people every year, the majority in low- and middle-income countries (LMICs).^1^ A few commercial products, notably tobacco, ultra-processed foods, and alcohol, are responsible for much of this health burden.^2^

 Taxation on products, restricting or banning advertising, restricting availability and altering product presentation – all designed to reduce sales or consumption of harmful products or ingredients – are identified by the World Health Organization (WHO) as the most cost-effective policy interventions, or ‘best buys,’^3^ for reducing NCDs. Unhealthy commodity industries (UCIs) which we define here as tobacco, ultra-processed foods (which includes sugar sweetened beverages and baby milk formula), alcohol and gambling, in particular, face a growing global risk to their operations, markets, and profits as more countries adopt these policies.^4,5^ Their response is to engage in corporate political activity (CPA) to avert immediate and future regulatory risks. CPA is a major obstacle to reducing the burden of NCDs globally.^6-9^

 The release of millions of internal tobacco industry (TI) documents through litigation in the 1990s provided unique insights into CPA, igniting a wave of research on TI behaviour.^10^ Systematic reviews used to make sense of this extensive literature, largely in the form of country case studies, show the TI consistently uses the same strategies across time and place.^6,11^ This in turn enabled the development of evidence-informed taxonomies of TI CPA^6,12^ which have been used to successfully predict and counter the TI’s influence. The taxonomies’ subsequent application to the ultra-processed food,^13,14^ alcohol,^15^ and gambling^16^ showed that these industries use very similar influence strategies.^17^ There is also evidence that a wider set of industries use similar practices to shape science.^18^ The fact that corporations operating in diverse industries have financial,^10^ operational^19^ and board level ties^20^ and use the services of the same public relations companies^17,19^ and also sometimes work collectively,^19^ may help explain these similarities in practice.

 To date however, despite this growing evidence that UCIs engage in similar practices, posing a formidable challenge to progressing public interest policy-making, there is no pan-industry conceptual framework of UCI CPA.

 We therefore aim to develop evidence-informed cross-industry taxonomies and a model applicable to a range of UCIs globally, aimed at a broad audience including academics, advocates, regulators, and policy professionals. Such a framework would aid comparative research and allow the public health and policy communities to systematically document, predict and effectively counter corporate strategies.

## Methods

 We focused on four UCIs: tobacco; alcohol; food (which here refers to ultra-processed foods, sugar-sweetened beverages and baby formula); gambling. We use the terms ‘UCI’ and ‘industry’ to refer to specific industries or groups of industries and the terms ‘corporate’ or ‘corporations’ when referring to a more generic group of corporate actors. Over the period December 2020 to September 2021, we used five complementary methods to develop and test our taxonomies: (1) critical interpretive synthesis review, (2) brief interviews, (3) expert co-author knowledge and recommended papers, (4) stakeholder workshops, and (5) testing. This is similar to Moher and colleagues’^21^ reporting guidelines development process, although we opted for a consultative model with our stakeholders rather than consensus-building. The study protocol was developed by four of the authors (KL, SU, AF, and ABG) who met frequently to discuss progress, analysis and emerging constructs. The wider team, including BH, MM, LH, and VT, met every six to eight weeks. These discussions and the findings from interviews, workshops and the testing were all integrated into the analysis and guided successive revisions and refinements of the taxonomies and the model.

###  Developing the Taxonomies

####  Data Sources

*Critical interpretive synthesis (CIS) review*^22^: The review aims were to explore (1) definitions, conceptualisations and schematic representations of UCI CPA and (2) instances of CPA. Search terms developed with help from a specialist librarian related to CPA, policy areas, industries and theories/concepts.^23^ We searched Scopus, Web of Science, Business Source Complete, and the International Bibliography of Social Sciences (from inception in 1951 to 18 December 2020). One researcher (KL or AF) screened the titles and abstracts and both assessed the full texts of the remaining records for inclusion. If consensus could not be reached, a third researcher adjudicated (SU). Only peer reviewed papers in English with an original conceptualisation of tobacco, alcohol, food and/or gambling industry CPA and/or reporting use of an original conceptualisation were included. In line with established CIS methodology, we did not conduct formal quality screening or exclude papers based on quality.^22^ We did, however, want to exclude opinion-based papers, so we required a methods section or other evidence that the work was based on empirical research for eligibility. The full search strategy and inclusion/exclusion criteria are in Table S1 (See Supplementary file 1). In line with the CIS method, we also included papers suggested by research team members.

*Brief interviews*: Two researchers (SU and KL) conducted seven online interviews (taking contemporaneous notes) with users of existing frameworks to elicit views on their usability and content and suggestions for the new taxonomies.

*Expert co-authors*: The research team had expertise across the four industries and had published extensively in this area, including developing and using the frameworks and papers included in this study.

####  Analysis

*Theoretical orientation*: Our approach was critical and interpretive, based on a social constructivist ontology in which we conceptualised policy-making as a complex adaptive system^24^ mediated by power relations and discourses. Our work was informed by: Stone’s^25^ concept of policy as narrative where actors compose ‘stories’ to define policy problems, attribute causes and blame, and formulate responses; Asen’s^26^ view of policy-making as a mediation between the rhetorical and the material; Russell and colleagues’^27^ view of policy-making as a rhetorical enterprise involving value-based argumentation. Our work was also loosely informed by Lukes’^28^ ‘three dimensions of power’ theory examining the behavioural/agentic and ideological/structural manifestations of power. Finally, we adopted some concepts from the positivist policy literature, such as agenda setting, venue shopping and policy cycles.^29^

*Analysis*: We combined CIS^22^ and constructivist grounded theory using conceptual coding, constant comparison within and across papers, memo writing and attention to divergent cases and discourse^30,31^; SU led the analysis. All included papers were analysed using the Atlas.ti 9 software; interview and workshop notes were analysed in Microsoft Word. Each paper was interrogated in two ways: (1) overall conceptualisation of CPA, figurative structures and terminology, comprehensiveness, analytic insights and use by others and (2) reported instances of CPA. We critically – and iteratively – examined and compared the different conceptualisations with each other and our previous work and inductively categorised instances of CPA to develop the model and taxonomies. Two researchers (SU and KL) double coded three papers and discussed and resolved coding differences.

###  Testing the Taxonomies

 We conducted two online stakeholder workshops with academics, advocates and policy professionals with knowledge or experience of UCI CPA to elicit views on the emergent taxonomies and their usability. Small group testing, where the emerging taxonomies were populated using CPA vignettes, was followed by whole group discussion. The workshops were recorded to aid analysis, but not transcribed. Finally, we conducted a review of the empirical CPA literature (Table S2, Supplementary file 1) to purposively select case-study papers that drew on in-depth primary data on one of the four industries against which the taxonomies could be tested. For this testing, two researchers (KL and AF) individually coded three papers and double coded one paper to ensure coding alignment.

## Results

 We report our results in the following order: the review; interviews and testing; the taxonomies; strengths and weaknesses of industry CPA; the CPA Model.

###  The Review 

**Figure 1:**
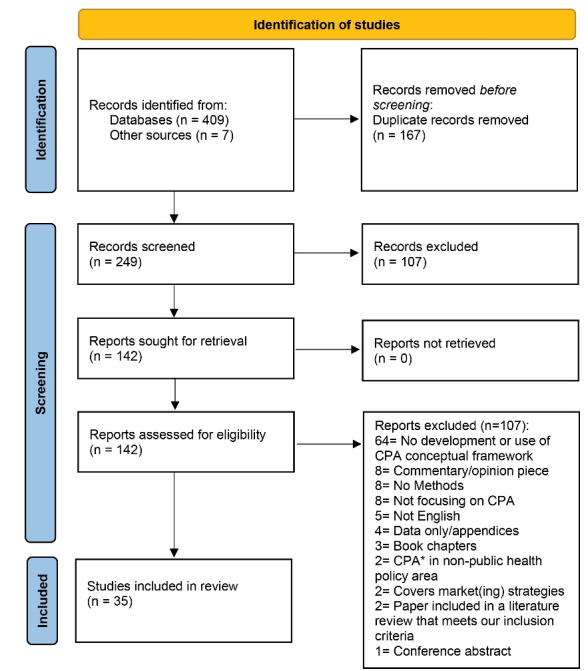
The PRISMA Flow Chart. Abbreviations: PRISMA, Preferred Reporting Items for Systematic Reviews and Meta-Analyses; CPA, corporate political activity.

 We identified 249 records for screening and included 35 studies: 11 contained an original conceptualisation of tobacco, alcohol or food industry CPA and 24 reported use of one of these original frameworks^6,12,13,18,32-38^ (Figure 1). All bar two^32,37^ included a methods section. Of the two, one paper^32^ contributed considerably to our understanding of CPA, referred to a literature review in ‘authors’ contribution’ and was extensively and consistently referenced despite not having a methods section and the other^37^ reported in its abstract that it was based on ‘publications’ and offered a novel conceptualisation. We noted that methods reporting in many papers was inadequate with scant details.

###  Existing Conceptualisations of Corporate Political Activity 

 The 11 conceptual frameworks varied in theoretical approach, methods, policies, industries, geographies, and structure^6,12,13,18,32-38^ (Table 1). One paper^33^ drew on concept mapping; all others were based on a form of literature review, with three^6,12,36^ using systematic reviews. All but two covered general CPA; one paper focused on specific issues around trade and investment agreements^38^ and another on science and use of science in policy.^18^ Most did not include a formal definition of CPA but two^13,34^ referred to the definition in a 1984 paper by Baysinger^39^ and one^35^ to the CPA paper by Hillman and Hitt,^40^ both defining CPA as attempts to shape public policy in ways that favour business interests. All but two^35,37^ paid attention to long-term (eg, forming relationships) as well as short-term manifestations (eg, blocking policies) of CPA. Two papers^32,38^ considered the impact of macro drivers of CPA, such as the political economy of neoliberal capitalism and corporate-friendly regulatory regimes. All but one^34^ paid attention to both the actions of corporate actors and their narratives, with the majority (9) using the concept of ‘framing’ in examining narratives.

**Table 1:** Papers Presenting an Original Conceptual Framework (n = 11)

**Papers**	**Industry**	**Policy**	**Geography**	**Jurisdiction**	**Theoretical Approach**	**Methods/Data**	**Analysis**
Trochim et al 2003^33^	Tobacco	Tobacco control programme evaluation	HIC-US	State (multiple)	None reported	Concept mapping	Thematic + statistical
Savell et al 2014^6^	Tobacco	Marketing	Global	Not specified	Hillman and Hitt, Resource dependence/market exchange 1999 (*business*)	Systematic review	Not reported – thematic analysis?
Mialon et al 2015^34,a^	Food	Mixed	Global	Not specified	None reported	Narrative review + grey literature	Not reported
Ulucanlar et al 2016^12,b^	Tobacco	Mixed	Global	Not specified	Critical approach; expansion and containment (*politics*)	Interpretive analysis of 2 systematic reviews	Constructivist grounded theory
Scott et al 2017^35^	Food	Reformulation	Global + US	National	None reported	Literature review + documents (consultation submissions)	Frames & narratives + inductive coding
Mialon et al 2018^13,c^	Food	Labelling	HIC-France	National + Regional (EU)	None reported	Literature review	Not reported
McCambridge et al 2018^36^	Alcohol	Mixed	Global	National + Supranational	None reported	Systematic review	Inductive thematic analysis
Madureira Lima and Galea 2018^32^	Mixed	Mixed	Not reported	Not specified	Steven Lukes, Power: a radical view, 2005 (*sociology*)	Not reported (‘Review’ mentioned in text)	Not reported
Capewell and Lloyd-Williams 2018^37^	Food	Mixed	Global	Not specified	None reported	Not reported (‘Publications’ mentioned in abstract)	Not reported
Milsom et al 2021^38^	Mixed	International trade	Global	National + Supranational	Fuchs and Lederer, framework of business power 2007 (*politics*) + Hall, 3-I framework, 1997 (*policy*)	Realist review	Not reported
Legg et al 2021^18^	Mixed	Does not examine policy. Examines corporate influence on science and its use in policy	Global	N/A	None reported	Scoping review and interpretive synthesis	Deductive coding and inductive thematic analysis

Abbreviations: HIC, high-income country; EU, European Union; N/A, not available. ^a^In turn based on Savell 2014. ^b^In turn based on Smith et al 2013 and Savell et al 2014. ^c^In turn based on Mialon et al 2015 & Ulucanlar et al 2016 (See Figure S1).

 Of the 11 conceptualisations, five were structured/presented as ‘taxonomies’^6,12,13,34,35^; two^32,38^ used theories of power to organise their analysis schematically; one^37^ was a ‘mnemonic’ encapsulating industry CPA in an easy-to-remember acronym; one^36^ used a simpler classification system; one^33^ was a very detailed list combined with a cluster map of frequencies.

 Of the five taxonomies, one^35^ (food) had a limited number of categories within an accompanying diagram. The other four (in food and tobacco) were closely related: two on tobacco^6,12^ were based on systematic reviews and led to the development of the other two on food^13,34^ with minor modifications (Figure S1). Three of the four^6,12,13^ distinguished explicitly between actions and narratives. In line with the original two,^6,12^ all four adopted a tabular, multi-level structure, going from the more general to the more specific, commonly using terminology such as ‘strategies,’ ‘tactics,’ ‘practices,’ and ‘arguments.’ These four taxonomies with tabulated details were the only frameworks within our dataset that had been used by other researchers, featuring collectively in the 24 review papers (Figure S1).

###  Papers Reporting Use of the Original Frameworks 

**Figure 2:**
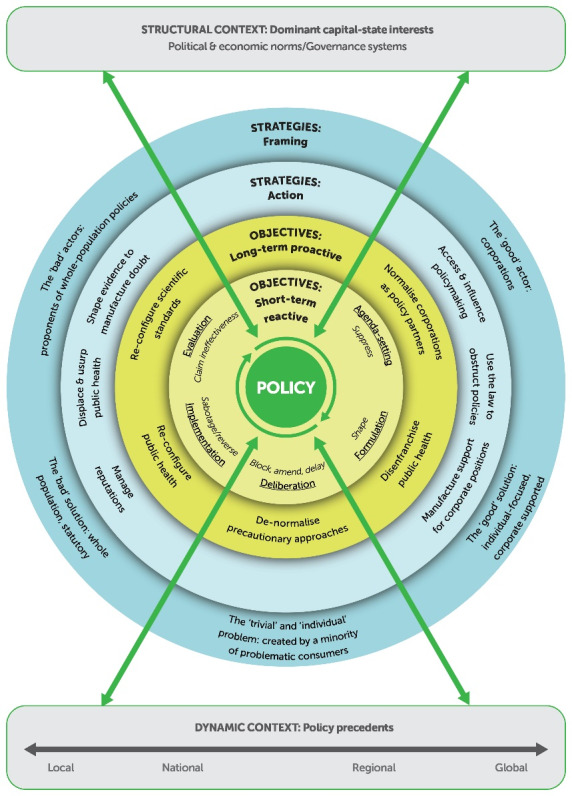
The Corporate Political Activity Model.

 The review of 24 papers^7,15,16,41-61^ (Table 2) that used one of the four taxonomies showed that all four were applicable and useful for examining the same industry in different geographical and policy contexts, with two^6,12^ also being used to study a different industry (Figure 2). Users of all taxonomies introduced changes or additional practices or arguments based on their data.^7,15,44,48,59^ In the case of alcohol industry influence on road safety policy,^15^ Ulucanlar and colleagues’ dystopia idea, where industry predicts that policies will have catastrophic consequences, was not relevant; instead, the alcohol industry adopted an active harm-reduction approach, supporting interventions with little impact on sales such as education campaigns. In applying Savell et al^6^ to the gambling industry,^16^ the authors found the separation of actions and narrative led to some ‘significant overlap’ and used only the actions framework with some modifications while a related paper by Martino and colleagues^62^ used only the narrative framework.

**Table 2:** Papers Reporting Use of the Original Frameworks (n = 24)

**Papers**	**Industry**	**Policy**	**Geography**	**Jurisdiction**	**Theoretical Approach**	**Methods/Data**	**Analysis**
Stillman et al 2008^41^	Tobacco	No specific policy – developing TI tracking capacity	LMICs-South East Asia	National	Trochim et al 2003	Structured expert opinion	Thematic + statistical analysis
Savell et al 2016^7^	Alcohol	Marketing	Global	Not specified	Savell et al 2014	Systematic review	Narrative synthesis Deductive coding to framework + inductive coding
Mialon et al 2016^42^	Food	Mixed	HIC-Australia	National	Mialon et al 2015	Digital (website) data	Deductive coding to framework + inductive coding
Mialon and Mialon 2017^43^	Food	Mixed	HIC-France	National	Mialon et al 2015	Digital (website) data	Deductive coding to framework
Tselengidis and Östergen 2019^44^	Food	Sugar tax	EU	Supranational	Mialon et al 2015	Digital (website) data	Deductive coding to framework + inductive content analysis
Jaichuen et al 2018^45^	Food	Mixed	LMIC-Thailand	National	Mialon et al 2015	Digital data/paper document review + interviews	Deductive coding to framework + inductive coding
Hancock et al 2018^16^	Gambling	TV advertising	HIC-Australia	National	Savell et al 2014	Document review (consultation submissions)	Deductive coding to framework + inductive coding
Mialon and da Silva Gomes 2019^46^	Food	Mixed	LMICs – South America + Caribbean	National	Mialon et al 2015	Digital (website) data	Deductive coding to framework
Oliveira da Silva et al 2019^47^	Tobacco	Additives	LMIC-Brazil	National	Ulucanlar et al 2016	Literature review + documents+ websites	Deductive coding to framework
Paixao & Mialon 2019^48^	Alcohol	Mixed	HIC-Portugal	National	Ulucanlar et al 2016 + Savell et al 2015	Digital (website + social media) data	Deductive coding to framework
Egbe et al 2019^49^	Tobacco	Mixed	LMIC-Nigeria	National	Ulucanlar et al 2016	Interviews + media reports + documents	Not reported
Tanrikulu et al 2020^50^	Food (baby)	Dietary guidelines	HIC-US	National	Ulucanlar et al 2016 + Mialon et al 2018	Digital (website + social media) data	Deductive coding to framework
Ojeda et al 2020^51^	Food (sugary drinks)	Mixed	LMIC-Mexico	National	Mialon et al 2015	Digital (website + social media) data + traditional media + interviews	Deductive coding to framework
Oliveira da Silva et al 2020^52^	Tobacco	Mixed	LMIC-Brazil	National	Ulucanlar et al -2016	Literature review + industry internal documents + digital (website) data	Narrative synthesis + deductive coding to framework
Mialon et al 2021^53^	Food	Labelling	LMIC-Colombia	National	Mialon et al 2018	Digital (websites, social media) data + interviews	Deductive coding to framework
Mialon et al 2020^54^	Food	Mixed	LMIC-South Africa	National	Mialon et al 2018	Digital (websites, social media) data	Deductive coding to framework
Mialon et al 2020^55^	Food	Mixed	LMIC-Colombia	National	Mialon et al 2018	Digital (website) data + interviews	Deductive coding to framework
Wood et al 2020^56^	Food (sugary drinks)	Mixed	LMIC-US	National	Mialon et al 2015	Digital communications (emails between industry and academics)	Deductive coding to framework + thematic content analysis
Abdool Karim et al 2020^57^	Food (sugary drinks)	Taxation	LMIC-South Africa	National	Ulucanlar et al 2016 + Mialon et al 2018	Digital documents (consultation submissions)	Deductive coding to framework
Hoe et al 2020^15^	Alcohol	Road safety	Mainly HICs – US, Canada	Not specified	Ulucanlar et al 2016	Mapping literature review + documents + interviews	Deductive coding to framework + inductive coding
Bhatta et al 2020^58^	Tobacco	Mixed	LMIC-Nepal	National	Ulucanlar et al 2016	Media reports + documents + interviews	Deductive coding to framework
Vandenbrink et al 2020^59^	Food	Mixed	HIC-Canada	National	Mialon et al 2015	Digital (website) data	Deductive coding to framework + inductive content analysis
Stafford et al 2020^60^	Alcohol	Mixed	HIC-Australia	State + Federal	Ulucanlar et al 2016	Documents (consultation submissions)	Documentary content analysis
Matthes et al 2021^61^	Tobacco	Mixed	LMICs-multiple	National	Ulucanlar et al 2016	Interviews	Deductive coding to framework + inductive coding

Abbreviations: HIC, high-income country; LMIC, low- and middle-income country; EU, European Union.

###  Interviews, Stakeholder Workshops and Testing Against the Literature

 Interviews with seven users of taxonomies, who had experience in tobacco, alcohol and food, indicated that the existing taxonomies were useful, but could be improved. Overlap between categories, where a practice could potentially be assigned to more than one strategy, was identified as a common problem. However, during stakeholder workshops (9 participants each), it was suggested that some overlap was inevitable as corporate actions were often directed at multiple strategic ends and close examination of the data and judgement were needed in categorising these. Another issue was the specificity of corporate arguments in one taxonomy^12^ which reduced their applicability to different contexts. Workshop participants suggested that the new taxonomies should be visually and linguistically simple, include well-established rhetorical devices (eg, ‘nanny state’), have fewer levels and broader categories but also include some detail and examples for clarity, and be applicable to all levels of governance including global organisations. This feedback led to a major revision.

 For the final testing of the taxonomies, we purposively selected seven papers,^63-69^ two for each industry except for gambling where we only identified one paper. The papers included a mix of policies and geographical settings; six were at the national level while one covered both state and federal jurisdictional levels. We populated the taxonomies with data from the papers to explore their fit to different industries and settings. This process showed that they were fit for purpose, but a number of new practices and arguments were added.

###  The Proposed New Taxonomies

 First, we offer a synthesized and updated definition of CPA: *Practices to secure preferential treatment and/or prevent, shape, circumvent or undermine public policies in ways that further corporate interests. *In developing our taxonomies, we opted for the well-used tabular format, but drew on ideas from all the review papers. In line with interview and workshop findings, we sought to use simple, clear and neutral terminology and a balance between abstraction/parsimony and detail/clarity. In common with most papers, we distinguish between industry actions and frames and present separate but complementary taxonomies of each. Our framing taxonomy loosely follows Benford and Snow’s^70^ typology of ‘diagnostic,’ ‘prognostic’ and ‘motivational’ frames, but with the addition of ‘actors.’ It details how corporate actors construct and present meanings for social phenomena, often in ways amenable to their interests.^71,72^ Our taxonomy of ‘action strategies’ details the means by which corporations seek to secure outcomes that will ultimately lead to favourable policies, including by persuading policy-makers, the public and other businesses of the need to base decisions on industry frames. We suggest that, in most cases, users will need to pay attention to both taxonomies. For example, UCIs argue that a particular policy is illegal (framing) and may or may not take legal action against it (action); or they claim that the policy is not evidence-based (framing) and produce pseudo-scientific critique to discredit papers that support the policy (action). The taxonomies are also represented in our CPA model which contextualises them within socio-political and economic systems.

 Like most existing taxonomies, ours have two levels: (1) higher, overarching categories (frames or strategies) and (2) explanatory or constitutive categories (frame-supporting claims or mechanisms). A third column provides more illustrative detail. We have tried to ensure that categories are mutually exclusive; however, as noted widely in the literature, it is likely that some CPA instances will fit into more than one category, posing a challenge to users. We advise that users consider contextual information and objectives of the categorisation when populating the taxonomies. Furthermore, users are advised to treat the illustrations in the third column as a guide and not as prescriptive or exhaustive lists. We expect future applications will uncover many different examples that are not currently in the taxonomies, but that will be accommodated by the higher categories, while additional claims or mechanisms may also be identified. Here, we give an overview of each taxonomy section; empirical examples for each category with references to the dataset can be found in Table S5 (Supplementary file 2) and Table S6 (Supplementary file 3).

###  Taxonomy of Framing Strategies

 We propose that UCI framing is nested within an overarching and simplistic dichotomy: corporate intentions, values and actions are ‘good’; those of the proponents of industry-opposed policies are questionable or ‘bad.’ An inversion is implied, whereby corporations position themselves as representing and speaking for the public interest while they position those proposing the policy as damaging the public interest through incursions, restrictions and non-market interventions. UCIs use this simple ‘good/bad’ dichotomy to frame three key issues – the actors, the problem and the solutions – in ways that make strong, regulatory public health responses less politically acceptable and less likely to be implemented. The intended audiences of framing include other businesses, policy-makers, civil society organisations, and the public (Table 3).

**Table 3:** The Taxonomy of Framing Strategies

**Framing the Policy Space**	**Frame-Supporting Claims**	**Illustrations**
The ‘good’ actor: corporations	**F-GA1.** Businesses are legal entities	Corporations have a right to conduct their business and to trade and abide by laws and regulations.
	**F-GA2.** Industry is key economic actor	Corporations are engines of economic growth and future prosperity.
	**F-GA3. **Industry is part of the social fabric	Corporations are socially embedded in country/region and part of its history.
	**F-GA3.** Industry is legitimate policy actor	Corporations understand the need to tackle health issues, are reasonable and willing to enter into partnership with government.
		Corporations have expertise and information that government needs in making policy.
		Corporations need access to policy spaces and decision-makers because they are part of the solution.
	**F-GA4.** Industry is legitimate scientific actor	Corporations support evidence-based policy.
		Corporations are legitimate scientific actors and have expertise in the science of product health harms and solutions.
		Corporations are valuable educational resources to the public health community.
	**F-GA5.** Industry is champion of public health	Corporations are responsible, committed to prevention of NCDs and working to reduce health harms.
		Corporations support the proposed policy.
	**F-GAI6.** Industry is socially responsible	Corporations create welfare by investing in social and economic development and are concerned with social justice.
		Corporations are committed and essential to sustainable development.
	**F-GA7.** Industry is victim	Corporations are unfairly demonised.
The ‘bad’ actors: proponents of whole-population, statutory policies	**F-QA1.** Policy-makers who support unfavourable policies have questionable skills and motives	Policy-makers may have good intentions but are incompetent/misguided, offering policies that contradict existing policies, are ineffective, illegal or not in keeping with international norms and standards.
		Policy-makers are disingenuous, for example, they want to raise revenue, not protect the public’s health, or have a hidden agenda, for example to introduce restrictions on other products or industries (*slippery slope*).
		Policy-makers are authoritarian and want to control people’s lives (*nanny-state*).
	**F-QA2.** Public health community have questionable skills and motives	Scientists are incompetent or untrustworthy, engaging in bad scientific practices and promoting false or misleading findings.
		Scientists and advocates are ideologically motivated and have anti-industry/anti-free-enterprise agenda.
		Scientists and advocates are fanatical and want to control the lives of the reasonable/responsible majority.
The ‘trivial’ and ‘individual’ problem: created by a minority of consumers	**F-P1. **Health harms are not caused by Industry’s products/services	Industry’s products/ingredients/services are harmless or cause minimal problems.
		Industry’s products/ingredients have been misclassified/confused with other, genuinely harmful products/ingredients.
		Health problems have complex causes that cannot be traced to industry products or services alone.
		Industry’s products/services contribute to health, wellbeing and enjoyment of life.
		Industry’s products/services are aligned with cultural norms and practices and are used responsibly by the majority.
	**F-P2.** Health harms arise from consumption patterns of atypical minorities	Health harms result from individuals’ or sub-populations’ wrong or uninformed choices and irresponsible behaviours.
		Health harms result from cognitive problems or physical/mental health problems.
	**F-P3.** Health harms are exaggerated	Health harms only affect a minority and are exaggerated by the public health community.
		There are far more serious and urgent health problems that government should prioritise instead.
The acceptable, ‘good’ solution: individual-focused, corporate supported	**F-S1.** Solutions should target individuals, not whole populations	Solution is to help individuals or ‘problem’ sub-populations to change their consumption behaviours through information, health education and promotion.
		Solution is to ‘treat’ problematic consumption using targeted interventions and ‘harm reduction’ approaches.
	**F-S2.** Solutions should be self-regulatory & not disrupt business	Self-regulation and voluntary action by corporations (on advertising, marketing, labelling, etc) are more effective and more compatible with business operations.
The unacceptable, ‘bad’ solution: whole population, statutory	**F-NS1. **Policies are unnecessary & unacceptable	Policy is unnecessary because corporations are successfully self-regulating and initiating public health interventions.
		Existing regulation is sufficient and should be better enforced before new measures are introduced.
		Policy is disproportionate to the problem.
		Policy is out of line with global standards and other countries’ policies.
		Policy is regressive and discriminatory.
		Policy is not evidence-based.
	**F-NS2.** Policies/policy formulation contravene norms, rules & laws	Government has not sufficiently consulted industry or other groups.
		Government has failed to conduct comprehensive social and economic impact assessment.
		The body proposing regulation has no legal authority to do so.
		Policy is unconstitutional, impedes basic rights (eg, the right to free speech) and curtails basic freedoms of a legal business.
		Policy is illegal (eg, it violates terms of international trade and investment agreements).
	**F-NS3.** Policies will lead to losses for businesses, economy & society	Policy will be impossible to implement (cost)effectively.
		Policy implementation will increase administrative cost to governments.
		Policy will reduce competitiveness, innovation and investment and lead to business closures and job losses (especially among SMEs and also in associated sectors like farming).
		Negative impacts on business will affect the wider economy, reducing GDP.
		Policy will discourage foreign investment in the country.
		In LMICs, policy will impede economic development and make LMICs less competitive.
		Corporations will not be able to support or invest in social justice projects.
	**F-NS4.** Policy will fail & have perverse consequences	Policy will not work or has not worked elsewhere.
		Policy is a blunt or simplistic instrument and will not achieve nuanced change.
		Policy will cause confusion or fear.
		Policy will increase illicit trade and smuggling or encourage cross-border shopping.

Abbreviations: NCD, non-communicable disease; GDP, gross domestic product; LMICs, low- and middle-income countries; SMEs, small and medium enterprises.

*The good actor: corporations*. UCI corporate actors construct for themselves a range of identities that position them as key commercial entities with high levels of competence, integrity and foresight. Moving beyond business and the claim of positive economic contribution, they profess a concern with and claim expertise in public health, science and social justice. Crucially, they present themselves as legitimate policy partners to government, meriting extensive access to policy-makers and possessing unique and essential information. When necessary, they also claim victimhood, arguing policy-makers make the conduct of business difficult or demonise corporations.

*The bad actors: proponents of whole-population, statutory policies or regulations which industry opposes*. Corporate actors portray proponents of such policies – whether they are government departments/global organisations with public health duties or public health professionals/advocates – as ineffective, misguided or disingenuous. They accuse them of making incompetent policy choices, having a hidden agenda of wider prohibitions (‘slippery slope’), or acting as the ‘nanny state’ and interfering in peoples’ lives. Similarly, independent scientists whose findings counter industry interests are charged with scientific misconduct, providing inaccurate information, giving bad advice to policy-makers and authoritarianism. In other contexts, for example where policies or approaches favour corporate interests, UCIs may frame the relevant organisations (including global ones) and governments more positively, claiming an alignment with industry and even producing shared narratives.

*The trivial problem: created by a minority of consumers*. Central to UCIs’ framing of the public health problem is the suggestion that their products or services do not harm health – and can, on the contrary, contribute to it. Any health problems are caused by a minority of uninformed or irresponsible users, for example ‘problem’ drinkers or gamblers, with the public health community exaggerating the problem. However, this framing has become less convincing for tobacco, an addictive product whose harms are universally acknowledged; instead, the TI refers to the ‘public health benefits’ of smoke-free products and promotes its role in a ‘smoke-free future.’^73,74^ In the same vein, the alcohol and gambling industries are keen to differentiate their products/services from tobacco products, arguing that unlike tobacco, the former are not intrinsically harmful but can be if misused. Another, deflective, framing technique is to argue that governments are wasting time and resources on a trivial problem and ignoring more urgent/bigger problems.^75,76^

*The acceptable, ‘good,’ solution: individual-focused, corporate supported.* Having framed the public health problem in this way, corporate actors define the solution as targeted interventions aimed at individual behaviour change through, for example, education and information particularly for the ‘irresponsible user’ or ‘harm reduction’ approaches that also enable industry products to be seen as part of the solution. They posit self-regulation and voluntary codes as appropriate and cost-effective ways to align industry practices with the public interest and people’s health. These framings are designed to minimise impact on market transactions and profits.

*The unacceptable, ‘bad,’ solution: whole-population, statutory*. UCI actors reject statutory public health interventions that target product supply and availability and impact on whole populations (and corporate profits). They produce and publicise a suite of reasons for why proposed policies are unacceptable and likely to fail with significant negative consequences. These include policy redundancy, incoherence, irregularity or illegality and the arguments that policies are regressive, not evidence-based and too restrictive, infringing on personal freedoms. In terms of negative consequences, UCIs consistently present potential corporate losses as public catastrophes. Thus, potential business impacts such as implementation costs, loss of sales, revenue, competitiveness, return on investment, possible closures and industry-specific job losses are almost always linked to and interwoven with impacts on the wider economy such as business closures across other sectors (eg, farming, advertising, informal sector), unemployment, smaller gross domestic product (GDP), reduced tax revenue, reduced foreign investment and slowed development for LMICs, and loss of industry investment in social justice projects or competitive sports. Meanwhile, UCI actors tend to remain silent on potential impacts on share prices, dividends and, sometimes, sales, all primary concerns for corporations but less likely to produce public sympathy or alarm. Another claim is that policies will fail or have failed elsewhere and will lead to perverse consequences such as confusing consumers. In some cases, especially in tobacco and alcohol, UCIs also argue that the policy will increase illicit trade and smuggling (despite, for example, the TI engaging in these^77^). The gambling industry argues that domestic restrictions will encourage people to seek (illegal) off-shore gambling sites.^78^

###  Taxonomy of Action Strategies

 Corporations take a wide range of actions to persuade policy-makers and the public of the legitimacy and value of their framings. We identified six strategies of which four target the central domains of policy-making: policy-makers and policy processes; the law; evidence; support from diverse sections of society. A fifth targets how public health is conceived and delivered on the ground and aims to shape these in line with corporate interests. The last, managing reputations, is a cross-cutting strategy that permeates and facilitates all the others. Importantly, individual strategies and practices can both overlap and work synergistically, enhancing their effectiveness. While we have teased them out into separate categories to create the taxonomy, in real life they often represent composite actions or chains of events. The following is an example: corporations pay for research (shape evidence to manufacture doubt); issue press releases about the findings and put forward experts to the media (manufacture public support for industry position); and take research summaries to meetings with policy-makers (access and influence policy-making) (Table 4).

**Table 4:** The Taxonomy of Action Strategies

**Action Strategy**	**Mechanisms**	**Example Practices**
Access and influence policy-making	**A-P1.** Access policy-makers and policy spaces	*Give incentives*: provide finances, resources, hospitality and gifts to politicians, officials, political parties, election campaigns, government departments, politicians’ selected charities, global organisations; give bribes.
		*Make threats*: threaten policy-making bodies at all levels with loss of corporate investment, corporate relocation or job and revenue losses.
		*Use the ‘revolving door’ and create conflicts of interest*: secure membership of or control policy-making bodies, working/technical/ advisory groups and committees, public-private economic and policy forums, national trade and conference delegations; conversely, recruit ex-officials/politicians to industry positions.
		*Seek regulatory capture*: a form of corporate incursion into government with corporate representatives and their interests playing a central role in regulatory body/government decision-making.
		*Access standard-setting fora:* use corporate consultants (scientific/health and safety) to access and enter into dialogue with standard-setting bodies.
	**A-P2.** Attempt to influence policy processes and outcomes	*Gather intelligence*: collect information on policies and politicians to inform lobbying.
		*Deliver policy*: offer dialogue and expertise or to take responsibility for designing and drafting policy.
		*Lobby the executive:* seek relationships and direct contact with relevant policy-makers to shape policy processes and secure industry-favourable outcomes.
		*Lobby the legislature: *use legislators to influence the introduction, advancement or modification of legislation and to deliver corporate-friendly testimonies.
		*Lobby through internal leverage*: seek to influence and oppose health departments/regulators via other departments or committees such as for business, trade, agriculture.
		*Use written consultation submissions*: imply authoritativeness and consensus, misreport research, overwhelm the process by large volume of submissions.
		*Use administrative barriers to undermine adopted policy*: seek to have implementation assigned to hostile/apathetic agencies or divert policy-mandated funds to other/less effective tasks and purposes.
		*Engage in non-compliance*: counter policy with promotions and discounts, fail to implement policy, use arguments/falsehoods and product availability to encourage non-compliance by businesses and the public.
	**A-P3.** Manage policy venues	*Use venue shifting*: ensure legislation occurs at regulatory jurisdictions more favourable to industry.
		*Use pre-emption at local, national and global levels:* use the authority of higher-jurisdiction bodies to constrain public health policy-making at lower jurisdictions.
Use the law to obstruct policies	**A-L1.** Use legal challenges to policy pre- and post-adoption	*Threaten/take legal action*: make (often false) claims of illegality under domestic laws or international trade and investment agreements and use countries to bring dispute cases to WTO against other countries’ policies.
		*Create ‘regulatory chill’*: use (pending) legal threats and action in other countries as precedents to deter or shape new policies.
	**A-L2.** Use the law to undermine policy-making/public health community	*Interfere with institutions*: attempt to remove powers from regulatory body; lobby judges/lawyers to influence proceedings.
		*Obstruct public health campaigners*: threaten or use legal action or injunctions to stop health advocates’ campaigns.
Manufacture public support for corporate positions	**A-S1.** Coordinate and manage industry strategies	*Conduct professionally managed campaigns*: engage consultants and legal, public relations and market research companies to manage strategies and to amplify corporate messages.
	**A-S2.** Form business alliances	*Joining forces with directly affected businesses*: coordinate strategies and share resources with other manufacturers, supply chain businesses, trade associations, employee organisations.
		*Secure support of indirectly affected businesses*: recruit as allies other industries, eg, hospitality, packaging, printing, advertising, media (both large companies and SMEs) and other sectors, eg, farming, sports and chambers of commerce.
	**A-S3.** Secure support beyond business	*Form alliances with key individuals and organisations*: buy and engineer support of influential individuals/experts, civil society organisations and foreign governments through payments/donations/help, false statements about policy and claims to represent disparate interests.
	**A-S4. **Fabricate allies	*Create front groups and others: *set up front groups, astroturf,^a^ SAPROs and others (eg, think-tanks) to use as campaign tools.
	**A-S5.** Operate through third parties	*Use allies (A-S2, A-S3, A-S4) to enact industry campaigns: *direct all forms of allies to engage in campaign activities including recruiting other allies, producing and disseminating information, media advocacy, responding to policy consultations, lobbying, initiating legal action and agitating on behalf of corporations.
		*Create impression of independence: *use varying degrees of concealment and opaqueness to hide links between allies (S2, S3, S4) and corporations to render their messages, actions and evidence more credible and acceptable.
	**A-S6.** Maximise corporate - favourable media content	*Access media through financial ties and relationships*: direct ownership, board membership, funding, relationships with and training and payments to journalists.
		*Access media by providing content: *advertising, advertorials, paid for content, press releases, arguing principle of ‘balanced’ reporting.
Shape evidence to manufacture doubt	**A-E1.** Undermine and marginalise unfavourable research/information	*Produce pseudo-scientific critique*: criticise (independent) research unfavourable to corporations using unachievable evidentiary standards and non-rigorous methods.
		*Misrepresent evidence*: misreport, ‘cherry-pick’ and misinterpret research and information unfavourable to corporations.
		*Marginalise unfavourable evidence*: reduce visibility and representation of (independent) research and information in the body of evidence by ignoring it or blocking publication.
		*Hide evidence*: hide unfavourable evidence produced or funded by corporations.
		*Misrepresent scientific norms*: over-emphasize complexity, uncertainty and disagreement among researchers.
	**A-E2.** Produce or sponsor favourable research/ information	*Create parallel scientific literatures*: produce or commission external institutions and scientists to create a self-referential body of alternative research that contradicts the international research literature.
		*Create information materials*: produce diverse materials targeting a variety of audiences to promote evidence favourable to corporations.
		*Promote falsehoods: *present false or inaccurate information in materials produced and in public discourse.
	**A-E3.** Amplify and blend corporate-favourable evidence into public record and discourse	*Promote favourable evidence*: widely disseminate favourable research and information using a variety of media and input from business and civil society allies.
		*(Self)-reference: *cite corporate-supporting and corporate-produced studies widely in (peer-reviewed) journal articles and other information sources.
		*Engage in questionable citation practices*: cite inaccessible, unpublished, unverifiable or non-peer-reviewed evidence.
		*Participate at scientific events: *use legitimate scientific platforms to showcase corporate-sponsored research, using independent scientists.
Displace and usurp public health	**PH1.** Undermine the rationale for statutory policies on corporate practices	*Seek policy substitution: *initiate self-regulation and voluntary codes relating to, for example, labelling, ‘conscious’ advertising, ‘responsible’ marketing, reformulation, etc, to prevent binding regulation.
	**PH2**. Deliver individual-level interventions	*Normalise less effective interventions*: use SAPROs, CSR and partnerships with NGOs, professional organisations, governments and global organisations to contribute to funding, planning, delivery and evaluation of life-style information and education interventions that also emphasize responsible consumption.
		*Divert attention to secondary issues*: deliver interventions that do not impact on the sale of products (eg, in obesity, interventions designed to encourage exercise instead of changing consumption of food).
	**PH3. **Promote ‘harm reduction’ as public health goal	*Develop ‘reduced harm’ products: *narrow the focus of interventions and market ‘healthier’ versions of products (eg, Diet Coke, e-cigarettes or low-alcohol drink), nutraceuticals, etc, as substitutes.
	**PH4**. Deliver education and training to public health professionals	*Provide public education*: produce educational materials, books, guidelines and organise workshops for health professionals in partnership with civil society, patient and professional organisations, government departments and global organisations.
	**PH5**. Weaken the public health community	*Fragment the public health community*: create divisions (‘extremists’ and ‘moderates’), distract or overwhelm the public health community.
		*Monitor and intimidate opponents:* infiltrate/monitor public health advocacy groups and independent researchers, threaten to withdraw support, intimidate individuals.
Manage reputations to corporate advantage	**R1.** Repair and nurture corporate reputations	*Highlight CSR and good deeds*: conduct CSR and philanthropy, providing funds and sponsorship to a variety of causes including (public) health organisations.
		*Substitute for weak government*: use operational scale and resources to deliver welfare and other services to populations.
		*Seek respectability by association*: publicly associate with respected individuals and organisations.
	**R2**. Discredit public health community	*Attack and defame:* defame public health researchers, advocates and organisations through attacks on their work and personal integrity.

Abbreviations: SME, small and medium enterprises; SAPROs, social aspect public relations organisations; CSR, corporate social responsibility; NGOs, non-governmental organisations; WTO, World Trade Organization. ^a^Astroturf: fake grassroots organisations.

*Access and influence policy-making*. A prime corporate strategy is to infiltrate the centre of policy-making – governments at all levels, global organisations and regulatory agencies – in order to proactively shape, delay, or stop policies. Access mechanisms include incentives (money, resources, hospitality, gifts, bribes) and, conversely, threats (industry pulling out of operations or investments). Corporations use revolving door practices to offer current and former politicians and officials positions within industry and secure roles for their staff within policy-making bodies, creating conflict of interest conditions within the policy space and sometimes leading to ‘regulatory capture’ (especially in LMICs) where regulatory and policy-making bodies are effectively beholden to corporations and no longer able to take independent and public interest decisions. Additionally, they seek to influence standard setting bodies and standards that have regulatory implications, sometimes through science and health and safety companies. Influence is also sought through gathering and presenting information/evidence, offering help, conducting various forms of lobbying, and participating in formal consultations. Two important mechanisms of influence are (1) venue shifting: transferring policy-making to politically more favourable jurisdictions where industry preferred outcomes are more likely, and (2) preemption: introducing less restrictive regulation at higher jurisdictions to foreclose stronger public health legislative possibilities at lower jurisdictions, sometimes for many years. Once a policy is adopted, corporations may attempt to undermine implementation through the use of administrative barriers or non-compliance.

*Use the law to obstruct policies*. Corporate actors try to stop policies by threatening or taking legal action under domestic and international laws or by supporting countries to initiate dispute procedures at global bodies like the World Trade Organization (Table S3, Supplementary file 1). Cases may be unlikely to succeed, but are initiated to delay policy implementation and to create a deterrent to other countries and future policy-making, known as ‘regulatory chill,’ especially in under-resourced LMICs. Industry also uses the law to interfere with policy-making bodies and remove their powers, influence judges and obstruct public health campaigners through (threat of) injunctions.

*Manufacture support for industry position*. To complement direct involvement, corporations try to give the impression of favourable public opinion through a wide variety of alliances. First, the various industry actors work to mitigate normally competitive relationships and to pool resources and ideas so they can speak in unison, often with input from public relations and other consultancies. Support is secured from indirectly affected peripheral businesses and other sectors – advertising or hospitality sectors, for example. Beyond business, public support is engineered by recruiting a wide range of civil society organisations (professional, consumer, patient, community), key individuals and foreign governments, using payments and false narratives where there is no interest convergence. Further, UCIs fabricate allies, setting up and/or funding a variety of industry-supporting organisations which can take the form of third parties, front groups or astroturf organisations.^79^ Allies add value to corporate campaigns, amplifying industry framings in (social) media and undertaking lobbying, litigation and agitation while keeping their links with corporations hidden or obscured. In this way, they create the impression that they are independent and that a very large segment of society supports the industry position. The use and manipulation of (social) media are critical in achieving this and UCIs use money and relationships (sometimes called ‘media capture’) as well as advertisements and promoted content to maximise favourable coverage.

*Shape evidence to manufacture doubt*. UCI corporate actors also try to create the impression that the health harms of their products and services, and the effectiveness of regulatory action are genuinely contested, despite the weight of evidence demonstrating both. They undermine studiesthat report harms or policy effectiveness/legality through pseudo-scientific and unscientific critique. More proactively, they create a parallel scientific literatureusing their own scientists and commissioning others to produce contradictory evidence on health harms and policy effectiveness, as well as to promote industry products or policy alternatives. They complement this with a very large body of non-scientific information, often containing falsehoods or half-truths, to be used as lobbying materials aimed at policy-makers, the media, the public, professionals and civil society organisations. The key to creating doubt is to amplify corporate-favourable research and information and to blend these into the body of evidence and public discourse, removing corporations’ fingerprints in the process through a variety of practices and the use of the diverse allies outlined above.

*Displace and usurp public health*. An increasingly important strategy is to create an apparent redundancy for regulation through policy substitution, or self-regulation and voluntary codes, as well as to shape public health provision. Corporations thus contribute to the funding, planning, delivery and evaluation of services through a variety of organisational forms and partnerships with public bodies (sometimes referred to as public-private partnerships). These are often less effective interventions with less impact on sales. The appropriation of the concept of ‘harm reduction,’ an established approach in drug addiction,^80-82^ enables corporations to narrow the focus of policies and to create new markets for ‘reduced harm’ products. UCI actors promote this weaker version of public health through providing education materials and activities to public health professionals. Finally, they engage in negative action to fragment the public health community by creating divisions or through monitoring and intimidation.

*Manage reputations to corporations’ advantage*. Although tobacco is an outlier, many UCI corporations suffer from a reputational deficit in the public health arena owing to their exclusive focus on profit maximisation. So they engage in corporate social responsibility (CSR) and “good deeds” and, particularly in under-resourced settings or countries, present themselves as a substitute for government, using their operational scale and resources to become involved in poverty alleviation, crisis management, law enforcement, etc. They also seek respectability by association, appearing at academic events, publicly declaring links with respected scientists, national and international organisations and government agencies and even using their logos on industry information materials. Conversely, they seek to undermine the reputation of researchers, advocates and organisations by attacking and defaming them through (social) media, blogs, correspondence, freedom of information requests, data access requests and official complaints.

 We now offer an analysis of the strengths and weaknesses of corporate strategies.

###  Strengths and Weaknesses of Corporate Strategies

 The CPA literature generally points to the well-resourced, well-organised and well-executed nature of CPA. These are features that we have also identified. At the same time, however, we have elicited weaknesses in corporate strategising, an understanding of which may be useful to public health advocates and policy-makers (Table S4, Supplementary file 1).

###  Strengths

 Extensive *financial resources* – or ‘heaps of money’^37^ – allow corporations to bankroll their various activities. The ability to *cooperate and coordinate* with other corporations and industries, and to speak with a unified voice is an obvious advantage, although there are exceptions to this.^11,16,36^ Well organised and often professionally managed campaigns produce homogeneous, widely circulated messages. Creating *synergy* – combining multiple strategies and practices for greater impact – is also a key strength.^36^ As shown in our framing taxonomy, *taking on multiple identities *– scientist, public health expert, policy-maker, development economist, consumer, citizen – allows UCI actors to create the impression of broad support but also to ‘colonise’ and shape different social domains. *Ubiquity *allowsthese actors to have presence in all potential policy venues and situations where influence may be exerted. We identify a further strength that we term ‘*hyper-adaptability,’ *defined as *a continuous ability to adapt to conditions and contexts by altering positions and methods*. Underlined by pragmatism and manifested as contradictions, hyper-adaptability enables corporations to use their strategies in different cultural, social, political, economic and jurisdictional settings and at different time points by selecting strategically from the menu of claims and practices and tailoring their narratives and actions to the cultural zeitgeist.^83^ One example is their approach to the transferability of evidence between settings. Where evidence from other countries is unfavourable to UCIs’ interests (eg, showing product harms or policy effectiveness),^57,61^ corporate actors argue the evidence cannot be valid elsewhere; where that evidence serves their interests (often evidence they have produced), they promote it, claiming it is relevant.^53^ Depending on context, UCIs try to stymie policies using two contradictory arguments: that population-level policies are too extensive and heavy-handed^36,44^ and that they are too simplistic.^13,36,60^ They give rhetorical support to evidence-based policy-making^36,56,60^ while misrepresenting high quality independent research and promoting alternative evidence, often its own, of poor quality and misleading.^12,33,57,60^ Corporations’ actions generally appear collaborative and constructive, for example offering help with policy-making, but, as the action taxonomy makes clear, when necessary, they engage in hostile and destructive action, for example issuing threats to governments. They encourage high-income countries (HICs) to counter policies of LMICs and vice versa^38^; they form alliances with chambers of commerce and businesses^58,60^ as well as with trade unions.^58,44^ Finally, their practices are on a continuum of visibility, ranging from the covert (bribery, undermining advocates, front groups) through opaque (lobbying, sponsoring research, directing allies’ activities) to overt (media campaigns).

###  Weaknesses

*Hyper-adaptability* can also be understood as a weakness, as it exposes the incoherence of corporate messaging and actions. Furthermore, notwithstanding hyper-adaptability, the core strategies are *highly predictable*, showing consistency over time and place and across industries, constituting a ‘play-book’ used not just by the industry sectors we examine here, but more broadly, including fossil fuels.^84-86^ Corporate actors make ample use of *easily fact-checked falsehoods*. Many corporate alliances represent ‘grafted common interests’^87^ or ‘stitching’^88^ and veil over a *dissonance of interests *between corporations and their allies. Occasionally, businesses *compete and clash* publicly over policies. A more subtle but crucial weakness is the *caricaturised accounts of social life *offered by corporate actors, where everything they represent and do is good and everything opponents represent and do are bad, for example: proposed policy is utterly bad, has no redeeming features and will have catastrophic consequences; only corporations (not the public health community) has the public’s interest at heart and the expertise to address the health problems. Finally, UCI actors’ *reluctance to publicly acknowledge obvious risks to themselves*, for example loss of sales, points to the dishonesty of their messaging. Awareness of the predictability of corporate actions, aided by tools such as the current taxonomies, will provide an opportunity to pre-empt them; exposing the inconsistencies and incoherence in corporate narratives will expose much of it as propaganda.

###  Contextual Variation

####  Corporate Political Activity Model

 We have presented our detailed taxonomies of UCI political strategies and their strengths and weaknesses. We are conscious, however, that these strategies are embedded within a wide nexus of economic, political, scientific, cultural, and social systems that can facilitate or hinder corporate strategies and success. Our analysis also focused on these wider phenomena and we present here the CPA model (Figure 2). The model captures the systemic nature of corporate influence on public policy and underlines the impact of context.

 The model first highlights that the strategies detailed so far (both the framing and action strategies in the outer circles of the model) are directed towards a range of objectives: short-term/reactive objectives aimed at solving specific policy ‘problems’ and long-term/proactive objectives directed at creating an enduring corporate-friendly policy environment. In the short term, corporate actors seek to influence all the stages of the policy cycle^29^: suppress public health concerns and keep them out of public discourse (agenda setting); contribute to shaping policy (formulation); block, substitute or amend policy to weaken it (deliberation/adoption); sabotage policy by preventing, delaying, undermining or reversing it (implementation); present policy as failed and ineffective so it will not be implemented elsewhere (evaluation). In the long term, they seek to: normalise industry as a policy partner; disenfranchise the public health community; re-configure and constrain public health as a non-regulatory, individual-focused practice in which corporations are essential; de-normalise precautionary approaches to narrow policy scope; re-configure scientific standards to make it more difficult to demonstrate both the need for and effectiveness of policy. Both the framing and action strategies are used eclectically to achieve these goals as the model shows. Second, the model draws attention to two types of context: structural and dynamic. The structural context refers to the dominant political and economic system and norms and stage of development where the policy is being made. The integration of capital and state and the dominance of their interconnected interests from national to global level in most settings constitute a corporate-enabling structural eco-system with political, economic and regulatory manifestations.^89^ These include, for example, neoliberalism as the global political/economic norm, the focus on GDP growth, global trade and investment rules that privilege economic exchange over public health and corporate-friendly regulatory regimes.^89^ Many corporate strategies are thus made possible by and benefit from an underlying receptivity of the state and society. At an empirical, dynamic level, policy outcomes at a variety of settings (local, national, regional, and global) provide important precedents for and shape the policy context for other settings. These precedents define the boundaries around political acceptability and financial costs of policy, for example when corporate actors claim policy has failed or initiate legal action against it, making other governments unwilling to pursue it.

 We suggest that an illustration of the impact of structural and dynamic contexts can be found in the more aggressive and possibly more successful CPA strategies corporations deploy in LMICs.^38,57,61^ The contextual factors that account for this include: the importance of LMIC markets to UCIs as HIC markets shrink and as LMIC economies and populations expand; the urgent goal of economic development in LMICs including investment (which can involve external pressure for trade and investment liberalisation including in products harmful to health) and dependence on exports for revenue; fewer technical, financial and legal resources of LMIC governments; greater power differential between governments and transnational corporations; less developed democratic institutions and traditions such as a free press and civil society advocacy.

 The CPA model thus goes beyond specifying the different strategies corporations use by offering an explanation for *why* industry actors use these strategies and *how* their use can be facilitated by invisible or unproblematised social and political norms and arrangements.

## Discussion

 In this study, we have sought to de-construct CPA into its micro elements (taxonomies of framing and action strategies) and also offer a macro-level view (the model), a holistic approach that we hope scholars, advocates and policy-makers will find useful. The CPA model recognises the complex, contingent and generative nature of policy-making and locates it within much broader social, political, economic, and cultural structures and empirical contexts that impact on it. Our taxonomies distinguish between framing strategies that define the policy actors, the public health problem and the appropriate solutions in ways that promote corporate goals, and action strategies designed to persuade policy actors of the legitimacy and value of these framings as guides to decision-making. We have highlighted the strengths and weaknesses of corporate strategising as a starting point to developing counter strategies.

 Our model and taxonomies are based on peer-reviewed data on four UCIs, systematic qualitative analysis, insights from expert co-authors and input from a stakeholder group of advocates, academics and policy professionals, who, between them, had experience of a range of industries (including all four covered here), countries and jurisdictional levels. However, our literature searches were restricted to peer-reviewed papers that developed or used a conceptual framework and it is likely that we missed some forms of CPA reported in papers without a conceptual focus or in the grey literature. Our sample had more papers relating to the food industry and only two for gambling (but was more evenly distributed across HICs and LMICs). Nevertheless, the majority of claims and practices were used by all four industries (though as noted, we had more limited evidence for gambling), suggesting that our taxonomies are widely applicable. Our use of reported data interpreted by other researchers enabled access to a much larger body of evidence than primary research would have allowed. Finally, reducing social complexity to the tabular format of a taxonomy was challenging, necessitating some compromise, for instance, simplification and artificial demarcations.

 Our evidence confirmed that all four UCIs employed very similar practices. It is also increasingly clear that mining,^90^ pharmaceuticals,^91,92^ fossil fuels^86^ and other industries^84,93^ also deploy similar CPA strategies. Policy influence by corporations in non-democratic states is less well understood, but there is some evidence that it follows similar patterns.^94^ As we have shown and as others have also highlighted,^95-97^ corporations are agile and opportunistic political actors who draw on both ideational (narrative) and material (resource-based) sources of power. Using multiple voices, they co-opt the fields of policy-making, science production, public health and public opinion, ultimately making it difficult to disentangle the propaganda from the genuine, rendering corporate-sponsored values, norms, standards and definitions dominant.^89,98^ Through the use of combined strategies and in particular the practice of preemption, they remove communities,’ countries’ and global organisations’ right to legislate to protect health, creating a dangerous democratic deficit. In deploying these strategies, corporations are enabled by a global, consolidated and networked base of corporate institutional power - which can even dwarf state power on occasion.^89,99^ In most countries, this power results in a pro-business agenda in which corporate objectives are assumed to be synonymous with the public good^100,101^ and neoliberal values are internalised. The ideological allegiance to free-markets and the aversion to regulation means that politicians are often willing to accommodate business objectives at the expense of the public interest.^102-104^ It also limits what policies may be considered or adopted.^105^

 As both human and planetary health come under increasing threats from the way capitalism is currently organised and in particular the single-minded pursuit of GDP growth,^106,107^ there is an urgent need for policy-makers, advocates and scientists to fully understand the nature and implications of CPA. This means understanding CPA not as an ordinary and legitimate phenomenon in participatory democracy but as a corruption of democracy and an illegitimate colonisation of both the public and the private spheres.^108^ As corporations become increasingly integrated into political decision-making and public deliberation, they become more than one stakeholder group among many and act instead as powerful entities that ‘attempt to use the power of government to advance private ends.’^40^

 Effective strategies to counter CPA and protect public policy from harmful corporate strategies are urgently needed to reverse the growing burden of NCDs on world populations. Notwithstanding Article 5.3 of the Framework Convention on Tobacco Control,^109^ work to identify and evaluate the best approaches is in its infancy.^110^ Here, we suggest some potential directions for this important work based on our taxonomies and model, although we recognise that this can only be a partial view of a much wider field of study.

 Our work highlights the predictable nature of UCI CPA and our model and taxonomies can therefore be used to develop simple tools to track, effectively expose and counter it. Such tools can be used both in the acute policy window to help ensure passage of specific public health policies and to monitor ongoing CPA. This surveillance of CPA can play an essential role in increasing transparency, addressing the democratic deficit in policy-making and ensuring that appropriate and timely counter-actions are taken. As such there is a case for institutionalising it into national public health surveillance systems as occurs for environmental pollution and vectors of communicable diseases.

 However, as lessons from tobacco control highlight, more sustainable approaches require us to understand and address the long-term strategies of corporations^111^ and our model can again be used here. It highlights, for example, corporate actors’ longer-term focus on normalisation, on reconfiguring scientific standards and on nudging policy-making towards risk-based rather than precautionary approaches. Public health must seek to redress such efforts, through denormalisation for example, and prevent their spread by being alert to corporate efforts to globalise such approaches to policy-making.^89^ This would, in turn, be enabled by more structural approaches including, for example, a global instrument modelled on Article 5.3^109^ which would seek to prevent corporations’ disproportionate access to policy spaces and increase transparency,^20,54,112^ and a statutory commitment to public health representation in trade and investment negotiations.^113^ It is clear, however, that Article 5.3 is not a panacea; the TI has used its power to adapt its influence strategies in light of 5.3 and is now increasingly operating via third parties and investing in ever more elaborate normalisation campaigns.^111,114^ This then requires a more systems-based approach^17,115^ that pays attention to how power circulates within political, economic and scientific systems^116^ and addresses the sources of power available to corporations. Adequate taxation systems to address extensive tax avoidance^117^ and the use of a tax on corporations to fund independent research, preventing their ability to use science as a political tool,^18^ are two examples. Governments and civil society can also make better use of their power.^118^

 Our findings call for a research and advocacy agenda that combines expertise, resources and insights across industry sectors and, at the same time, pays close attention to structural factors, in particular neo-liberal capitalism as the fundamental cause of health harms^89,119,120^ and focuses on solutions to CPA. While there is always scope for more case studies that track corporate strategies at different geographical, jurisdictional, political and sociocultural settings, with representation from LMICs,^119^ particularly on the gambling industry, above all studies must shift to focus on potential solutions. A significant research gap is the perspectives and practices of policy-makers at national, regional and supranational levels with regard to CPA; researchers must find creative and sensitive ways to engage the policy community in ethnographic studies that will shed light on this relative ‘black box.’^53^ Finally, researchers should engage with policy-makers, the public and civil society organisations through (social) media to transfer their knowledge on how corporations influence policy-making and harm people’s health and to expose the faulty logic implied in corporate narratives that UCIs can be *both* ‘problem makers’ *and* ‘problem solvers.’^64^

## Acknowledgements

 We thank the anonymous reviewers for their insightful comments and our stakeholder participants (those who are named below and those who wished to remain anonymous) for helping with the development of the CPA taxonomies: Safura Abdoolkarim, Petronell Kruger, Catherine Egbe, Pascal Diethelm, Connie Hoe, Anca Toma, Maisha Hutton, Stella Bialous, Nason Maani, Erin McEvoy, Camila Maranha, Andre Silva, Joana Madureira Lima, Adam Bertscher, Katherine Severi, Maik Duennbier, Paula Johns, Niamh Fitzgerald, and Samantha Thomas.

## Ethical issues

 This research was approved by University of Bath Research Ethics Approval Committee for Health (REACH ref: EP 20/21 012).

## Competing interests

 Authors declare that they have no competing interests.

## Funding

 This work was supported by Bloomberg Philanthropies’ Stopping Tobacco Organisations and Products (STOP) funding (https://www.bloomberg.org/) and by the UK Prevention Research Partnership (MR/S037519/1), which is funded by the British Heart Foundation, Cancer Research UK, Chief Scientist Office of the Scottish Government Health and Social Care Directorates, Engineering and Physical Sciences Research Council, Economic and Social Research Council, Health and Social Care Research and Development Division (Welsh Government), Medical Research Council, National Institute for Health Research, Natural Environment Research Council, Public Health Agency (Northern Ireland), The Health Foundation and Wellcome. The funders had no role in the design and conduct of the study, data collection, data management, data analysis and interpretation and preparation, review and approval of the manuscript.

## Supplementary files

Supplementary file 1 contains Tables S1-S4 and Figure S1.

Supplementary file 2 contains Table S5.

Supplementary file 3 contains Table S6.
