# Supplementary material for: Corporate Political Activity: Taxonomies and Model of Corporate Influence on Public Policy
Source: Int J Health Policy Manag. 2023 Jun 6;12:7292. doi: 10.34172/ijhpm.2023.7292 (PMC10462073; doi:10.34172/ijhpm.2023.7292)
Supplement: Supplementary file 1 — contains Tables S1-S4 and Figure S1. [file ijhpm-12-7292-s001.pdf]

Article title: Corporate Political Activity: Taxonomies and Model of Corporate Influence on Public Policy

Journal name: International Journal of Health Policy and Management (IJHPM)

Authors' information: Selda Ulucanlar[1], Kathrin Lauber[2], Alice Fabbri[1], Ben Hawkins[3], Melissa Mialon[4], Linda Hancock[5], Viroj Tangcharoensathien[6], Anna B. Gilmore[1]*

[1]Tobacco Control Research Group (TCRG), Department for Health, University of Bath, Bath, UK.

[2]School of Social and Political Science, University of Edinburgh, Edinburgh, UK.

[3]MRC Epidemiology Unit, University of Cambridge, Cambridge, UK.

[4]Trinity Business School, Trinity College Dublin, Dublin, Ireland.

[5]Alfred Deakin Institute, Deakin University, Melbourne, VIC, Australia.

[6]International Health Policy Programme, Ministry of Public Health, Nonthaburi, Thailand.

(*Corresponding author: Email: a.gilmore@bath.ac.uk)

Citation: Ulucanlar S, Lauber K, Fabbri A, et al. Corporate political activity: taxonomies and model of corporate influence on public policy. Int J Health Policy Manag. 2023;12:7292. doi:10.34172/ijhpm.2023.7292

Supplementary file 1

Table S1: Critical interpretive synthesis review: additional information

Search Log			
Date	Database	Search string	Nr of results
17 Dec 2020	Scopus	(TITLE-ABS-KEY ("industry interfere*" OR "corporate political" OR "industry involvement" OR "industry political" OR "industry tactic*" OR "industry strateg*" OR "company tactic*" OR "company strateg*" OR "corporate influenc*" OR "industry influenc*") OR TITLE-ABS-KEY ("corporate interference" OR "corporate involvement" OR "corporate strateg*") AND TITLE-ABS-KEY (polic* OR "better regulation" OR trade) AND TITLE-ABS-KEY (food OR beverage OR drink OR alcohol OR tobacco OR gambling) AND TITLE-ABS-KEY (concept* OR model OR taxonom* OR framework OR theor*) AND DOCTYPE (ar OR re)	157
18 Dec 2020	Web of Science	**TOPIC:**("industry interfere*" OR "corporate political" OR "industry involvement" OR "industry political" OR "industry tactic*" OR "industry strateg*" OR "company tactic*" OR "company strateg*" OR "corporate influenc*" OR "industry influenc*" OR "corporate interference" OR "corporate involvement" OR "corporate strateg*") *AND* **TOPIC:**(polic* OR "better regulation" OR trade) *AND* **TOPIC:** (food OR beverage OR drink OR alcohol OR tobacco OR gambling) *AND***TOPIC:** (concept* OR model OR taxonom* OR framework OR theor*)	207<br>(129 removed as duplicates upon import)
18 Dec 2020	Business Source Complete	ALL IN ABSTRACT: ("industry interfere*" OR "corporate political" OR "industry involvement" OR "industry political" OR "industry tactic*" OR "industry strateg*" OR "company tactic*" OR "company strateg*" OR "corporate influenc*" OR "industry influenc*" OR "corporate interference" OR "corporate involvement" OR "corporate strateg*") AND (polic* OR "better regulation" OR trade) AND (food OR beverage OR drink OR alcohol OR tobacco OR gambling) AND (concept* OR model OR taxonom* OR framework OR theor*)	13<br>(11 removed as duplicates upon import)
18 Dec 2020	ProQuest/IBSS	noft("industry interfere*" OR "corporate political" OR "industry involvement" OR "industry political" OR "industry tactic*" OR "industry strateg*" OR "company tactic*" OR "company strateg*" OR "corporate influence" OR "industry influenc*" OR "corporate interference" OR "corporate involvement" OR "corporate strateg*" OR corporate tactic*) AND noft(polic* OR "better regulation" OR trade) AND noft(food OR beverage OR drink OR alcohol OR tobacco OR gambling) AND noft(concept* OR model OR taxonom* OR framework OR theor*)<br>• Additional limits: peer reviewed, full text - Source type: Scholarly Journals; Document type: Article; Language: English	32<br>(25 removed as duplicates upon import)
TOTAL (before removal of duplicates)			409

Table S2: Review of the empirical CPA literature: additional information

Search Log			
Date	Database	Search string	Nr of results
1 February 2021	Web of Science	**TOPIC:**("industry interfere*" OR "corporate political" OR "industry involvement" OR "industry political" OR "industry tactic*" OR "industry strateg*" OR "company tactic*" OR "company strateg*" OR "corporate influenc*" OR "industry influenc*" OR "corporate interference" OR "corporate involvement" OR "corporate strateg*") *AND* **TOPIC:**(polic* OR "better regulation" OR trade) *AND* **TOPIC:** (food OR beverage OR drink OR alcohol OR tobacco OR gambling)<br>**Refined by: DOCUMENT TYPES:** (ARTICLE OR REVIEW)	421
1 February 2021	Business Source Complete	ALL IN ABSTRACT: ("industry interfere*" OR "corporate political" OR "industry involvement" OR "industry political" OR "industry tactic*" OR "industry strateg*" OR "company tactic*" OR "company strateg*" OR "corporate influenc*" OR "industry influenc*" OR "corporate interference" OR "corporate involvement" OR "corporate strateg*") AND (polic* OR "better regulation" OR trade) AND (food OR beverage OR drink OR alcohol OR tobacco OR gambling)	32
1 February 2021	Scopus	(TITLE-ABS-KEY ("industry interfere*" OR "corporate political" OR "industry involvement" OR "industry political" OR "industry tactic*" OR "industry strateg*" OR "company tactic*" OR "company strateg*" OR "corporate influenc*" OR "industry influenc*" OR "corporate interference" OR "corporate involvement" OR "corporate strateg*") AND TITLE-ABS-KEY (polic* OR "better regulation" OR trade) AND TITLE-ABS-KEY (food OR beverage OR drink OR alcohol OR tobacco OR gambling)) AND (LIMIT-TO (DOCTYPE , "ar") OR LIMIT-TO (DOCTYPE , "re"))	385
1 February 2021	ProQuest/IBSS	noft("industry interfere*" OR "corporate political" OR "industry involvement" OR "industry political" OR "industry tactic*" OR "industry strateg*" OR "company tactic*" OR "company strateg*" OR "corporate influence" OR "industry influenc*" OR "corporate interference" OR "corporate involvement" OR "corporate strateg*" OR corporate tactic*) AND noft(polic* OR "better regulation" OR trade) AND noft(food OR beverage OR drink OR alcohol OR tobacco OR gambling)<br>- Additional limits: peer reviewed, full text - Source type: Scholarly Journals; Document type: Article; Language: English	133
TOTAL (before removal of duplicates)			971

**Purposive sampling criteria**
At least two papers for each of the four industries
Diversity in terms of: geographic location and type of public health policy
Case study: focus on specific policies in relation to at least one stage in the policy’s life: initial introduction, legislative debates, public consultation, implementation, evaluation.

Figure S1: The four CPA taxonomies and their use in the literature

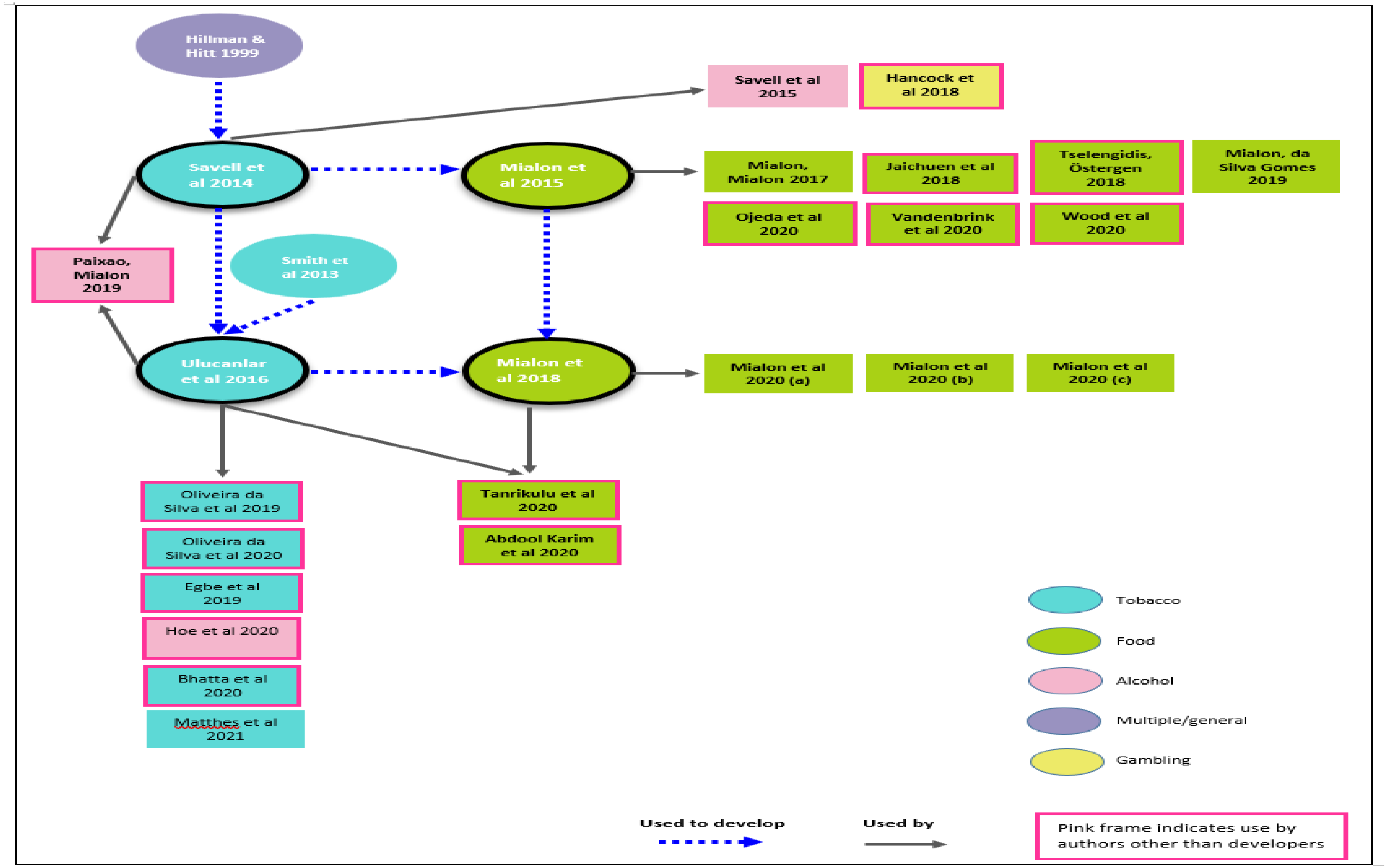

Table S3: Types and sources of legal challenges

Type of right implicated*	Arguments/Right invoked	Sources of legal challenges
Civil and political rights	Right to free speech	Domestic law; International law
	Right to property:<br>• Intellectual property such as trademarks impacted	Domestic law; International law
Socio-economic and cultural rights	Right to trade, including:<br>• Adding barriers to trade (and not legitimately meeting the necessity test);<br>• Undermines free competition;<br>• Undermines goal of harmonising trade laws	International law (International Trade and Investment Law)<br><br>Institutional procedures (e.g. World Trade Organisation)
	Policy in breach of legal business (legality challenge)	Domestic law; International law
Non-specified	Policy breaches individual rights	Domestic law; International law
	Unlawful lobbying & campaigning by public health advocates	Domestic law

* Civil and political rights are viewed as immediately realisable, while socio-economic and cultural rights are subject to progressive realisation.

Table S4: Strengths and weaknesses of industry CPA

**Strengths**	**Weaknesses**
• Financial resources<br>• Cooperation and coordination<br>• Synergy between strategies<br>• Multiple identities<br>• Ubiquity at policy spaces<br>• Hyper-adaptability	• Hyper-adaptability<br>• Predictability<br>• Easily fact-checked falsehoods<br>• Dissonance of interests with ‘allies’<br>• Corporate competition and discord<br>• Caricaturised accounts of social life
