# Supplementary material for: Corporate Political Activity: Taxonomies and Model of Corporate Influence on Public Policy
Source: Int J Health Policy Manag. 2023 Jun 6;12:7292. doi: 10.34172/ijhpm.2023.7292 (PMC10462073; doi:10.34172/ijhpm.2023.7292)
Supplement: Supplementary file 2 — contains Table S5. [file ijhpm-12-7292-s002.pdf]

Article title: Corporate Political Activity: Taxonomies and Model of Corporate Influence on Public Policy

Journal name: International Journal of Health Policy and Management (IJHPM)

Authors' information: Selda Ulucanlar[1], Kathrin Lauber[2], Alice Fabbri[1], Ben Hawkins[3], Melissa Mialon[4], Linda Hancock[5], Viroj Tangcharoensathien[6], Anna B. Gilmore[1]*

[1]Tobacco Control Research Group (TCRG), Department for Health, University of Bath, Bath, UK.

[2]School of Social and Political Science, University of Edinburgh, Edinburgh, UK.

[3]MRC Epidemiology Unit, University of Cambridge, Cambridge, UK.

[4]Trinity Business School, Trinity College Dublin, Dublin, Ireland.

[5]Alfred Deakin Institute, Deakin University, Melbourne, VIC, Australia.

[6]International Health Policy Programme, Ministry of Public Health, Nonthaburi, Thailand.

(*Corresponding author: Email: a.gilmore@bath.ac.uk)

Citation: Ulucanlar S, Lauber K, Fabbri A, et al. Corporate political activity: taxonomies and model of corporate influence on public policy. Int J Health Policy Manag. 2023;12:7292. doi:10.34172/ijhpm.2023.7292

Supplementary file 2

Table S5: The taxonomy of action strategies: examples from the dataset

ACTION STRATEGY	MECHANISMS	EXAMPLE PRACTICES	EMPIRICAL EXAMPLES
**Access and influence policymaking**	**A-P1**. Access policymakers and policy spaces	***Give incentives***: provide finances, resources, hospitality and gifts to politicians, officials, political parties, election campaigns, government departments, politicians' selected charities, global organisations; give bribes.	• In Australia, Coca Cola donated AUD 55,000 to the Australian Labour Party and to the Liberal Party of Australia for the financial year 2013-14. Woolworths' political contributions exceeded AUD 35,000 in 2014[42].<br>• In Brazil, the tobacco industry paid the equivalent of $15,000 to a member of the National Congress who introduce a bill aimed at cancelling the public consultation on a range of tobacco control legislation[52].
		Make threats: threaten policymaking bodies at all levels with loss of industry investment, industry relocation or job and revenue losses.	• In South Africa, faced with a sugar sweetened beverage tax, a beverage company said that (if the tax was approved) it would need to "reconsider its committed investments" in agriculture and agro-processing[57].<br>• At the EU, food companies threatened to withdraw investments if a sugar tax was approved[44].<br>• In Nepal, the US Chamber of Commerce, which had close ties with the tobacco industry and opposed tobacco control laws in that country, warned the government that legislation would 'negate foreign investment and invite instability'[58].
		Use the 'revolving door' & create conflicts of interest: secure membership of or control policymaking bodies, working/technical/ advisory groups and committees, public-private economic and policy forums, national trade and conference delegations; conversely, recruit ex-officials/politicians to industry positions.	• At the UN, special envoy for road safety was the president of the Federation Internationale de l'Automobile (FIA); FIA licences and sanctions Formula One, which receives sponsorship from Heineken[15].<br>• In Thailand, a relative of a food company's president was a former minister of commerce and a member of parliament[45].<br>• In the US, Nestle subsidiary Gerber was a member of the Department of Agriculture Centre for Nutrition Policy and Promotion's Nutrition Communicators Network[50].
		Seek regulatory capture: an extreme form of industry incursion into government with industry representatives playing a central role in regulatory body/government.	• In Colombia, the government and food industry interact with each other; members of large trade associations hold public sector positions[54].
		Access standard-setting: use industry consultants (scientific/health and safety) to access and enter into dialogue with standard-setting bodies.	• In Spain, the tobacco industry and ventilation standard setting agencies were linked via indoor air quality consultants, who conducted measurements of indoor air quality in buildings for the tobacco industry. One consultant often met with Philip Morris to update the company on ventilation issues. The company used its

			relationships with consultants to obtain confidential information from internal government meetings about building ventilation standards[68].
	**A-P2**. Attempt to influence policy processes and outcomes	***Gather intelligence***: collect information on policies and politicians to inform lobbying.	• In 2010, COP4 included discussion of developing guidelines on restricting the use of additives in tobacco products. Brazilian farmers protested against this outside the COP4 meeting, and tobacco industry-linked congressmen pressed to change the members of the Brazilian delegation to COP4. However, it emerged that a member of the Brazilian delegation was providing information from COP4 to the tobacco industry[47].<br>• The food industry in Thailand had representatives on government food agencies/committees and when a relevant policy was being planned, they would know about it early on[45].
		Deliver policy: offer dialogue and expertise or to take responsibility for designing and drafting policy.	• During negotiations for the Trans-Pacific Partnership Agreement and Transatlantic Trade and Investment Partnership, trade officials from the US and European Commission met privately with tobacco companies to discuss these agreements. In New Zealand, the food and beverage industry had greater direct access to decision makers about obesity and diabetes policy than other actor groups[38].<br>• In France, the dairy industry had relationships with the French government. The Conseil National de l'Alimentation (French National Food Council) provided input on policy – it was consulted about the definition of public food policy and provided expertise on questions[43].<br>• In Lesotho, Malawi, Uganda and Botswana, an alcohol company was responsible for drafting alcohol policy documents. These focused on self-regulatory measures, education campaigns, and directed responsibility for policy implementation to a National Alcohol Council, which included industry representatives[7].
		Lobby the executive: seek relationships and direct contact with relevant policy makers to shape policy processes and secure industry-favourable outcomes.	• In Nepal, following the Parliament's approval of the Tobacco Product (Control and Regulatory) Act, the tobacco industry lobbied politicians to prevent the bill from reaching the president for approval. The industry wanted to amend the bill[58].<br>• In Brazil, following publication of policy to ban additives in tobacco products, the tobacco industry pressured the Brazilian Health Regulatory Agency (ANVISA) to allow additives, and create exemptions, including menthol[47].
		Lobby the legislature: use legislators to influence the introduction, advancement or modification of legislation and to deliver pro-industry testimonies.	• In Nigeria, during debates on the National Tobacco Control Bills 2011 and 2012 in the National Assembly, legislators were visited by lobbyists and representatives of the tobacco industry[49].<br>• In Colombia, a politician reported that 'more than 60' lobbyists attended Congress to try and 'sink' a food labelling bill[53].

			• In Brazil, the proponent of a proposed policy to stop the results of a Public Consultation on banning tobacco additives had received campaign donations from tobacco companies[47].
		Lobby through internal leverage: seek to influence and oppose health departments/regulators via other departments or committees such as in business, trade, agriculture.	• In Nepal, the tobacco industry attempted to weaken and prevent implementation of 2011 tobacco control law via relationships with legislators. These aimed to create disagreements between government departments[58].<br>• In South Africa, a representative of the sugar industry explained to the media that their campaign against the introduction of a sugar tax benefited from interventions by government departments including trade and industry and forestry and fisheries[54].<br>• In Costa Rica, the president's office met with the Ministry of Economy, Industry, and Commerce (MEIC) during the public consultation of cigarette package health warning labels. The MEIC supported the tobacco industry's arguments, and argued that the labels would form technical barriers to trade. The MEIC sent a letter to the Health Ministry saying that the labels would need to meet technical regulations, including notification with the World Trade Organization Technical Barriers to Trade Agreement[65]
		Use written consultation submissions: imply authoritativeness and consensus, misreport research, overwhelm the process by large volume of submissions.	• Responses to the US 2015 Dietary Guidelines Advisory Committee reformulation consultation included 41 responses (out of 65 total responses) from food and beverage industry actors. Of 971 total responses to the whole DGAC online consultation, 286 came from food and beverage industry actors. 50 submission categorised as 'other' also came from industry partnerships or industry-funded groups[35].<br>• In Australia, gambling industry submissions to the 2013 Australian Parliamentary Committee Inquiry cited other companies and research favourable to their position in their submissions. They put their submissions on their company websites to share their arguments[16].<br>• For a consultation in Brazil on a tobacco display ban and new requirements for health warning labels, only 28 out of 140754 letters received by post/hand-delivered were effectively a contribution. Only 301 received by 1020 received by email were effectively contributions. The tobacco industry and supporters used printed forms to protest new policies – over 95% of participants identified themselves as tobacco growers[52].
		Use administrative barriers to sabotage adopted policy: seek to have implementation assigned to	• In Nepal, following Parliament's approval of the Tobacco Product (Control and Regulatory) Act, the tobacco industry lobbied politicians to prevent the bill from reaching the president for approval[58].

		hostile/apathetic agencies or divert policy-mandated funds to other/less effective tasks and purposes.	• In Brazil, a Federal Supreme Court injunction suspended a ban on tobacco product additives. The National Confederation of Industries questioned the competence of the Brazilian Health Regulatory Agency (ANVISA) to regulate tobacco products' additives[47].
		Choose non-compliance: counter policy with promotions and discounts, fail to implement policy, use arguments/falsehoods and product availability to encourage non-compliance by businesses and the public.	• In Nepal, the tobacco industry claimed that the ban on smoking in public places would violate the rights of smokers and be difficult to implement. Encouraging people and businesses to disobey the law[58].<br>• In Mexico, interviewees reported that to counteract new labelling and advertising laws and a sugar tax, industry changed the size of sugary drinks[51].
	**A-P3**. Manage policy venues	***Use venue shifting***: ensure legislation occurs at regulatory jurisdictions more favourable to industry.	• In Australia, the gambling industry chose to lobby the national parliament rather than state parliaments because the former is responsible for telecommunications legislation which impacts on gambling[16].<br>• In the UK, the alcohol industry engaged in venue shopping between the devolved Scottish and Westminster governments[36].
		Use pre-emption at local, national & global levels: use the authority of higher-jurisdiction bodies to constrain public health policymaking at lower jurisdictions.	• Our evidence for this came from review papers that identified this as a practice; we had no case-studies that provided specific examples. This is a practice that is relevant to federated structures of governance, such as the US. For examples in the US, the following website is very useful: https://grassrootschange.net/preemption-watch/. For information on global pre-emption, this paper is useful: Eric Crosbie, Arian Hatefi, Laura Schmidt, Emerging threats of global preemption to nutrition labelling, *Health Policy and Planning*, Volume 34, Issue 5, June 2019, Pages 401–402, https://doi.org/10.1093/heapol/czz045
**Use the law to obstruct policies**	**A-L1**. Use legal challenges to policy pre- & post-adoption	***Threaten/take legal action***: make (often false) claims of illegality under domestic laws or international trade and investment agreements and use countries to bring dispute cases to WTO against other countries' policies.	• In Thailand, tobacco companies claimed that the proposed cigarette ingredients disclosure legislation would violate the Trade Related Aspects of Intellectual Property Rights (TRIPS) agreement. In Canada and Australia, tobacco companies claimed that proposed plain packaging violated intellectual property right under TRIPS and NAFTA[38].<br>• In Scotland, the alcohol industry argued that proposed minimum unit pricing constitutes a technical barrier to trade[38].<br>• In Chile, food corporations challenged a labelling and marketing law at court[46].<br>• In Nepal, the tobacco industry filed a Supreme Court case arguing the pictorial health warnings law was unconstitutional, prohibited trade rights and violated trade agreements among others[58].

		Create 'regulatory chill': use (pending) legal threats and action in other countries as precedents to deter or shape new policies.	• In Canada, the alcohol industry argued that specific health warnings on bottles and cans would violate international trade law. This led to the policy not be adopted[38].<br>• Philip Morris used legal action against Uruguay over graphic cigarette warnings, and against Australia over plain packaging, to serve as deterrents for countries considering similar policies[32].<br>• In Thailand, interviewees reported that the food industry threatened the government with World Trade Organisations disputes in order to stop public health policies[45].
	**A-L2**. Use the law to undermine policymaking/public health community	***Interfere with institutions***: attempt to remove powers from regulatory body; lobby judges/lawyers to influence proceedings.	• In Brazil, the food industry questioned the timeline of the technical public consultation for front-of pack nutrition labelling. On the last day of the consultation, food industry actors lobbied the Court, resulting in the consultation submission deadline being extended[66].<br>• In Brazil, a tobacco industry friendly legislative proposal would have removed the authority of the main public health agency to regulate tobacco product additives, but this provision was removed from the proposal[47].<br>• In Nepal, the tobacco industry reportedly had financial links with the judges presiding over a public hearing on tobacco control legislation; as a consequence, this is thought to have delayed the hearing for 25 months[58].
		Obstruct public health campaigners: threaten or use legal action or injunctions to stop health advocates' campaigns.	• In Brazil, the tobacco industry secured an injunction against a public hearing on a law banning additives by arguing the venue was too small; the delayed hearing took place at a sports centre with 24,000 capacity with only 100 people[47].<br>• Interviewees in a LMIC reported that the tobacco industry tried to stop foreign funding of a tobacco control organisation[61].
**Manufacture support for industry position**	**A-S1.** Coordinate & manage industry strategies	***Conduct professionally managed campaigns***: engage consultants and legal, public relations and market research companies to manage strategies and to amplify industry messages.	• In Brazil, the tobacco industry paid for an advertising campaign against a ban on the display of tobacco products and changes to health warning labels. These aimed to amplify the message that the proposed policies would increase illicit trade, negatively impact farmers and small business, and negatively impact smokers' freedoms[52].<br>• In Spain, Philip Morris International used a public relations firm, Leo Burnett, to create a campaign designed to reduce the impact of 'unnecessary' tobacco-related regulation. Similarly, in 1995, British American Tobacco implemented its 'resocialisation of smoking' programme[68].<br>• As sugary drinks taxes are being introduced across the US, the sugary drinks companies and American Beverage Association are mobilising opposition by

			donating financially to media campaigns against the tax, creating fake opposition organisations, and funding local community groups[37].
	**A-S2**. Form business alliances	***<u>Joining forces with directly affected businesses</u>***: coordinate strategies and share resources with other manufacturers, supply chain businesses, trade associations, employee organisations.	• In South Africa, industry actors making submissions for a sugar tax policy, harmonised their arguments, endorsing and referencing other industry actors' submissions[57].<br>• In the USA, Nestle used 'internal constituency recruitment' in the baby food industry. Nestle was a member of industry-led organisations, including the Infant Nutrition Council (members included Abbott Nutrition, Perrigo Nutritional's and Reckitt Benckiser), and the Sustainable Food Policy Alliance (members include the US divisions of Danone, Mars and Unilever)[50].<br>• In Australia, gambling industry submissions to the 2013 Australian Parliamentary Committee Inquiry cited other companies and research favourable to their position in their submissions[16].
		<u>Secure support of indirectly affected businesses</u>: recruit as allies other industries, e.g. hospitality, packaging, printing, advertising, media (both large companies and SMEs[1]) and other sectors, e.g. farming, sports and chambers of commerce.	• In Finland, the alcohol industry's allies included sports federations, including the Finnish Ice Hockey Federation. This alliance enabled advertising at sporting events[69].<br>• In Australia, the gambling industry formed a common platform with media and sporting organisations[16].
	**A-S3**. Form alliances with key individuals & organisations	***<u>Secure support beyond business</u>***: buy and engineer support of influential individuals/experts, civil society organisations and foreign governments through payments/donations/help, false statements about policy and claims to represent disparate interests.	• In Mexico, the sugar-sweetened beverage industry influence policymaking via the Mexican Federation of Diabetes (received funding from food and sugar sweetened beverage industries) endorsing a book on hydration from Coca Cola. The opinion of the Mexican Federation of Diabetes was aligned with these companies and promoted their products[51].<br>• In Nigeria during the legislative process leading to the 2015 National Tobacco Control Bill, multiple groups, previously un-connected to the tobacco industry, promoted arguments which aligned with the industry's. For example, the African Liberty Organisation protested against the law during a public hearing. Prominent individuals, including past legislators and an ex-President, also supported the industry[49].
	**A-S4.** Fabricate allies	***<u>Create front groups and others:</u>*** set up front groups, astroturf[2], SAPROs[3] and others (e.g. think-tanks) to use as campaign tools.	• In Mexico, the sugar-sweetened beverage industry opposed public health measures on sugar-sweetened beverage tax increases, by creating the organisation MOVISA (Movement for a Healthy Life). This group included other food and sugar sweetened beverage industry-linked groups, and was developed by the industry following discussions about the sugar sweetened beverage tax increases[51].

			• In Costa Roca, following implementation of the 2012 tobacco control law, tobacco industry front groups (CACORE, Costa Rican Chamber of Restaurants; and CCH, Costa Rican Chamber of Hotels) submitted complaints to the Health Ministry. These suggested that they had each lost 25% of their revenues. However, evidence at the time suggested that 80% of employers lost no revenues[65].
	**A-S5**. Operate through third parties	***<u>Use allies (A-S2, A-S3, A-S4) to enact industry campaigns:</u>*** direct all forms of allies to engage in campaign activities including recruiting other allies, producing and disseminating information, media advocacy, responding to policy consultations, lobbying, initiating legal action and agitating on behalf of industry.	• In France, a policy proposal to require the use of 'Nutri-Score' (a labelling system for nutritional value) on advertisements was opposed by TV channels and media outlets via a joint letter to the government and ministries of health, agriculture, and education. The letter claimed that the policy would threaten their profits[13].<br>• In Nepal, the tobacco industry opposed the 2011 tobacco control law on the basis that it would cause job losses. Industry-linked front groups asked that politicians not implement the policy to protect its citizens' livelihoods[58].<br>• In Portugal, the brewers' association used a nutrition expert to make its case to the media when opposing alcohol policies[48].
		<u>Create impression of independence:</u> use varying degrees of concealment and opaqueness to hide links between allies (S2, S3, S4) and industry to render their messages, actions and evidence more credible and acceptable.	• In Colombia, a food industry front group (ILSI Nor-Andino, a branch of the International Life Science Institute) included Alpina, Coca-Cola, Kellogg, Mondelez, Nestle, Pepsico, Postobon, and Unilever in 2019. ILSI went on to collaborate with the Ministry of Health and universities, and influence policymaking, without disclosing its links to the food industry[53].<br>• In Nigeria during the legislative process leading to the 2015 National Tobacco Control Bill, the African Liberty Organisation for Development opposed the Bill during a public hearing. Prominent individuals, including past legislators and an ex-President, also supported the industry[49].
	**A-S6.** Maximise industry-favourable media content	***<u>Access media through financial ties & relationships</u>***: direct ownership, board membership, funding, relationships with and training and payments to journalists.	• In Thailand, a transnational soft drinks company donated drinking water to a newspaper company. The marketing manager of a fast food company donated food to the editorial department of a newspaper company. Food companies paid journalists to protect their company's image in their reporting[45].<br>• In Colombia, an economic group (Ardila Lulle Group) owns both a TV channel (RCN) and a beverage company (Postobon). Consequently, public health campaigns were censored on TV and radio[55].
		<u>Access media by providing content:</u> advertising, advertorials, paid for content, press releases, arguing principle of 'balanced' reporting.	• In LMICs, the tobacco industry paid think tanks, market research companies and professional service providers to produce inaccurate reports, which were then amplified by the media[61].<br>• In a LMIC, a journalist told researchers that their editor had asked them to print content provided by a tobacco company's public relations officer as a 'regular article'[61].

**Shape evidence to manufacture doubt**	**A-E1**. Undermine & marginalise unfavourable research/information	***Produce pseudo-scientific critique***: criticise (independent) research unfavourable to industry using unachievable evidentiary standards and non-rigorous methods.	• As part of the US' consultation on the 2015 Dietary Guidelines Advisory Committee, the food industry's submissions criticised evidence which linked their products to non-communicable diseases or obesity. They argued that the evidence was of low quality or insufficient quantity, and pointed out the uncertainty in the evidence base[35].<br>• In Australia, alcohol industry submissions to policy consultations between 2013 and 2017 focused on the need for an acceptable level of proven effectiveness for policies. They claimed that the current evidence was not of acceptable quality, and there should be a higher threshold for evidence quality[60].<br>• In South Africa, industry actors criticised the evidence used by the government and public health advocates to introduce a sugar sweetened beverage tax in 2016. The evidence behind the tax was criticised as being biased and not relevant to South Africa[57].
		Misrepresent evidence: misreport, 'cherry-pick' and misinterpret research and information unfavourable to industry.	• In Australia, the majority of alcohol industry submissions to policy consultations misused published evidence[60].<br>• In Australia, gambling industry submissions to the 2013 Australian Parliamentary Committee Inquiry presented evidence selectively and made inaccurate claims. The industry argued that: that most bets were made safely, not mentioning evidence of harms of 'moderate' and 'low-risk' gambling[16].<br>• In Ireland, food industry submissions to a consultation on a sugar sweetened beverage tax referred to a 2014 McKinsey Global report which listed taxation low down on a hierarchy of effective policy interventions. But the submissions neglected to mention measures higher up on the list, such as reducing the availability of high calorie food[64].
		Marginalise unfavourable evidence: reduce visibility and representation of (independent) research and information in the body of evidence by ignoring it or blocking publication.	• In Australia, the alcohol industry ignored evidence from national surveys and datasets regarding alcohol risks and harms while quoting evidence on reduced consumption[60].<br>• Another example can be found in: Brownell KD, Warner KE. The Perils of Ignoring History: Big Tobacco Played Dirty and Millions Died. How similar is Big Food? The Milbank Quarterly. 2009;87(1).
		Hide evidence: hide unfavourable evidence produced or funded by industry.	• Our evidence for this came from a review paper that identified this as a practice; we had no case-studies that provided specific examples. Information can be found in: Bero LA. Tobacco Industry Manipulation of Research. Late Lessons from Early Warnings: Science, Precaution, Innovation EA report number 1/2013: European Environmental Agency; 2013 (page 161).

		Misrepresent scientific norms: over-emphasize complexity, uncertainty and disagreement among researchers.	• In Ireland, the sugary drinks industry insisted on proof of causality between sugary drinks and adverse health outcomes in its submissions to the sugar tax consultation[64].
	**A-E2**. Produce or sponsor favourable research/information	***Create parallel scientific literatures***: produce or commission external institutions and scientists to create a self-referential body of alternative research that contradicts the international research literature.	• In Mexico, the sugar-sweetened beverage industry tried to influence decision making on the sugar-sweetened beverage tax by hiring academics to conduct biased studies and present them inaccurately (Ojeda et al, 2020).<br>• In South Africa, the alcohol industry commissioned a report on the economic impact of banning alcohol advertising. This was relied on in a presentation made by an advertising research consultancy (BMI Sports Info) and the alcohol industry at a consultation on the role of alcohol advertising in sport[63].
		Create information materials: produce diverse materials targeting a variety of audiences to promote industry favourable evidence.	• In Canada, a food association provided the public health agency with results from an unpublished survey supporting their position on food labelling[59].<br>• In Australia, Coca Cola and Nestle used information materials with extracts from poster presentations at scientific events to promote their products as healthy; a major food trade association re-tweeted a tweet promoting cereals as helping to lower risk of being overweight; Nestle provided information on physical activity on its website, 'Nestle Healthy Active Kids' with recipes promoting its products[42].<br>• In France, the dairy industry distributed free leaflets with dietary advice and posters for health professionals' waiting rooms[43].<br>• In South Africa, the alcohol industry asked a consultancy firm to prepare a report on the economic impact of banning alcohol advertising[63].
		Promote falsehoods: present false or inaccurate information in materials produced and in public discourse.	• In Brazil, only 10 of 127, 905 tobacco industry responses to a consultation on a proposed additives ban were a genuine contribution. These submissions used false addresses, false signatures and anonymous contributions[47].<br>• In Nigeria, during the process leading to ratification of the Framework Convention on Tobacco Control, British American Tobacco Nigeria claimed that it employed thousands of workers while its website showed that it only employed about 1000.After tobacco control advocates intervened publicised this, the company removed employment figures from its website[49].<br>• In Nepal, the tobacco industry argued that the proposed 90% pack health warnings was impossible to implement as it would require five pictures to be printed on packs; the truth was that the law would only require one of five rotating pictures to be printed[58].
	**A-E3**. Amplify & blend industry-	***Promote favourable evidence***: widely disseminate favourable research and information using a variety of media and input from business and civil society allies.	• In South Africa, submissions in response to a proposed sugar-sweetened beverage tax includes ones commissioned by the food industry (from the Rippe Lifestyle

	favourable evidence into public record & discourse		Institute funded by Kellogg and PepsiCo; from Hahn & Hahn, and the Glycemic Index Foundation of South Africa, Inc funded by Pioneer Foods)[57].<br>• In The US, Nestle conducted and promoted a large survey of parents and caregivers of infants and young children (FITS); the results were published in more than 50 peer-reviewed journal articles over 15 years and were also referenced in the American Academy of Paediatrics' handbook for paediatric nutrition[50].
		(Self)-reference: cite industry-supporting studies widely in (peer-reviewed) journal articles and other information sources.	• In Portugal, the Associação Nacional de Bebidas Espirituosas (ANEBE, National Association of Spirit Drinks) delivered a market study from a consultancy company to Parliament. This opposed a potential tax increase on alcoholic drinks. The alcohol industry heavily relied on this study in its lobbying against the proposed policy[48].<br>• In Brazil, the food industry widely cited two non-academic, non-peer-reviewed reports it had commissioned from industry-friendly organisations in its campaign against nutritional labels[66].
		Adopt questionable citation practices: cite inaccessible, unpublished, unverifiable or non-peer-reviewed evidence.	• In Canada, food and beverage industry documents used in reference to the 'Health Eating Strategy' in interactions between Health Canada and stakeholders, relied on citations, where a majority were not available online or available upon request. Some were also unpublished, not peer-reviewed, or industry-funded[59].<br>• In Brazil, a tobacco-industry funded report, published by the Getúlio Vargas Foundation (FGV), claimed that prohibiting additives would have no health benefits and would increase illicit trade. This report misrepresented its citations, cited authors with conflicts of interests, and made unfounded conclusions. The authors of the report provided no information on their credentials or conflicts of interests[47].
		Participate at scientific events: use legitimate scientific platforms to showcase industry-sponsored research, using independent scientists.	• In Portugal, representatives of the alcohol industry organised scientific conferences in partnership with the Portuguese Association of Nutrition. They invited researchers, and used their affiliations to claim credibility[48].<br>• In Australia, Coca Cola sponsored a session as part of the 2014 Nutrition Society of Australia Annual Scientific Meeting on weight loss maintenance. This followed a public consultation on a draft of the Australian Dietary Guidelines and Australian Guide to Healthy Eating[42].
	**PH1.** Undermine the rationale for statutory policies	***Seek policy substitution:*** initiate self-regulation and voluntary codes relating to, for example, labelling, 'conscious' advertising, 'responsible' marketing, reformulation, etc., to prevent binding regulation.	• In France, in response to the proposed policy of Nutri-Score, several food companies, including Coca Cola and Mars, developed an alternative front-of-pack nutrition system ('Evolved Nutrition Label')[13].

**Displace and usurp public health**	on industry practices		• In the European Union, in order to avoid legislation, the food and drink industries formed the EU Platform for Action on Diet, Physical Activity and Health which partners with the public sector to self-regulate on reformulation and marketing[44].<br>• In Uganda, Malawi, Lesotho and Botswana in 2009, alcohol policies reflected industry interests due to industry influence. These policies focused on industry involvement in policy development and self-regulation of advertising[38].<br>• In Australia, the gambling industry promoted non-regulatory initiatives on 'responsible gambling' and consumer education[16].
	**PH2**. Deliver individual-level interventions	***Normalise less effective interventions***: use SAPROs[3], CSR[4] and partnerships with NGOs[5], professional organisations, governments and global organisations to contribute to funding, planning, delivery and evaluation of life-style information and education interventions that also emphasize responsible consumption.	• The alcohol industry has delivered education programmes online. For example, SABMiller's www.TalkingAlcohol.com. In 2009, Diageo's 'Responsible Drinking Fund' claimed to have supported over 130 programmes focused on education and increased awareness[7].<br>• In Portugal, a trade association for spirit producers created an online tool for people to calculate alcohol concentration in their blood before driving[48].<br>• In Australia, a gambling company (Crowne Melbourne) in 2012 claimed that it was committed to providing responsible gambling. The ALH Group in 2014 claimed to be providing responsible gambling measures, including: self-exclusion, voluntary pre-commitment, and staff training to intervene with gamblers[67].
		Divert attention to secondary issues: deliver interventions that do not impact on the sale of products (e.g. in obesity, interventions designed to encourage exercise instead of changing consumption of food).	• In Portugal, the alcohol industry partnered with police and security forces through the SAPRO '100% Cool', awarding drivers with no alcohol detected on their breath, and delivered information on responsible drinking[48].<br>• The alcohol industry partners with public organisations to deliver road safety interventions which focus on prevention of drink-driving[15].<br>• In Thailand, the food industry supported physical activity programmes aimed at children and schools, including: 'Charity bowling', 'a walk rally to promote protection against osteoporosis', and 'junior football championship'[45].
	**PH3.** Promote 'harm reduction' as public health goal	***Develop 'reduced harm' products:*** narrow the focus of interventions and market 'healthier' versions of products (e.g. Diet Coke, e-cigarettes or low-alcohol drink), nutraceuticals, etc, as substitutes.	• In Mexico, the sugary drinks industry promoted no-calorie or reduced calorie drinks[51].<br>• In Canada, food companies promoted their voluntary decision to reduce trans-fat and reduced salt bread[59].<br>• In South Africa, Coca Cola SA and Pioneer Foods committed to reduced sugar products[57].
	**PH4**. Deliver education &	***Provide public education***: produce educational materials, books and guidelines and organise workshops for health professionals in partnership with civil society, patient and	• In Colombia, a local branch of the food industry sponsored International Life Science Institute presented a workshop on food labelling to undergraduates in

	training to public health professionals	professional organisations, government departments and global organisations.	nutrition and at a Colombian Association of Dietetics and Nutrition event (Mialon 2021).<br>• In Australia, McDonalds and Nestle provided education materials to schools, parents and the general public[53].<br>• In Mexico, Coca Cola linked with the Mexican Red Cross to provide nutrition workshops for health professionals[51].
	**PH5**. Weaken the public health community	***<u>Fragment the public health community</u>***: create divisions (‘extremists’ and ‘moderates’), distract or overwhelm the public health community.	• In Nigeria, during debates on the National Tobacco Control Bills 2011 and 2013, the tobacco industry tried to introduce bills to regulate the work of non-governmental organisations in attempts to weaken and destabilise tobacco control advocates[49].<br>• In the US, Philip Morris fragmented the anti-tobacco movement by dividing them into ‘extremists’ and ‘moderates’ and partnering with the latter[32].
		<u>Monitor & intimidating opponents:</u> infiltrate/monitor public health advocacy groups and independent researchers, threaten to withdraw support, intimidate individuals.	• In tobacco farming countries, the tobacco industry has intimidated tobacco control advocates by trying to remove their funding and making anonymous phone calls and correspondence[61].<br>• Public health professionals working on ultra-processed food and drink products (a researcher in Brazil, the direction of Nutrition policy at Mexico’s National Institute of Public Health, and the head of a consumer advocacy group in Colombia) received personal threats[46].
**Manage reputations to industry’s advantage**	**R1.** Repair & nurture industry reputation	***<u>Highlight CSR[4] & good deeds</u>***: conduct CSR and philanthropy, providing funds and sponsorship to a variety of causes including (public) health organisations.	• In South Africa, the South African Sugar Association (SASA) donated sugar to community food security programmes. SASA also donated support to tertiary education students via its “Sugar Industry Trust Fund for Education”[54].<br>• In Nigeria, British American Tobacco Nigeria trained the police force in enforcement of the tobacco control legislation[49].<br>• In France, Danone had involvement in the community, via programmes on nutrition, water, sanitation, and hygiene. It was also a partner of the French Red Cross and the French branch of the Ronald McDonald Foundation[43].
		<u>Substitute for weak government</u>: use operational scale and resources to deliver welfare and other services to populations.	• In lower- and middle-income countries, the tobacco industry has worked with public relations companies for activities, such as building health facilities and schools[61].<br>• In Colombia, the food industry partnered with the government and professional organisations to set up the Alliance for Child Nutrition to eliminate child malnutrition by 2030[53].
		<u>Seek respectability by association</u>: publicly associate with respected individuals and organisations.	• In the USA, Nestle provided charts on infant growth and development to medical professionals. These charts included the Nestle logo next to the US Centre for

			Disease Control and Prevention's (CDC) logo, to indicate endorsement by the CDC of Nestle's charts[50].<br>• In Colombia, Nestle had its nutrition programme for children validated by the Faculty of Nursing and Rehabilitation of the University of La Sabana[55].<br>• At the 2014 ICPAPH (International Congress on Physical Activity and Public Health), Coke managed to have a former Centre for Disease Control (US) scientist deliver its message about EIM (Exercise is Medicine, a Coke sponsored partnership)[56].
	**R2**. Discredit public health community	***Attack & defame:*** defame public health researchers, advocates and organisations through attacks on their work and personal integrity.	• In response to criticism of Coca Cola's sponsorship of the 2014 International Congress of Physical Activity and Public Health, a food industry representative referred to critics as "miscreants". It referred to a minority who were opposed to a majority of evidence-based researchers[56].<br>• In Brazil, the food industry attacked the regulatory agency ANVISA questioning its competence in assessing the available evidence, accusing it of using evidence[66].
