# Supplementary material for: Corporate Political Activity: Taxonomies and Model of Corporate Influence on Public Policy
Source: Int J Health Policy Manag. 2023 Jun 6;12:7292. doi: 10.34172/ijhpm.2023.7292 (PMC10462073; doi:10.34172/ijhpm.2023.7292)
Supplement: Supplementary file 3 — contains Table S6. [file ijhpm-12-7292-s003.pdf]

Article title: Corporate Political Activity: Taxonomies and Model of Corporate Influence on Public Policy

Journal name: International Journal of Health Policy and Management (IJHPM)

Authors' information: Selda Ulucanlar[1], Kathrin Lauber[2], Alice Fabbri[1], Ben Hawkins[3], Melissa Mialon[4], Linda Hancock[5], Viroj Tangcharoensathien[6], Anna B. Gilmore[1]*

[1]Tobacco Control Research Group (TCRG), Department for Health, University of Bath, Bath, UK.

[2]School of Social and Political Science, University of Edinburgh, Edinburgh, UK.

[3]MRC Epidemiology Unit, University of Cambridge, Cambridge, UK.

[4]Trinity Business School, Trinity College Dublin, Dublin, Ireland.

[5]Alfred Deakin Institute, Deakin University, Melbourne, VIC, Australia.

[6]International Health Policy Programme, Ministry of Public Health, Nonthaburi, Thailand.

(*Corresponding author: Email: a.gilmore@bath.ac.uk)

Citation: Ulucanlar S, Lauber K, Fabbri A, et al. Corporate political activity: taxonomies and model of corporate influence on public policy. Int J Health Policy Manag. 2023;12:7292. doi:10.34172/ijhpm.2023.7292

Supplementary file 3

Table S6: The taxonomy of framing strategies: examples from the dataset

FRAMING THE POLICY SPACE	FRAME-SUPPORTING CLAIMS	ILLUSTRATIONS	EMPIRICAL EXAMPLES
**The 'good' actor: industry**	**F-GA1.** Businesses are legal entities	Industry has a right to conduct its business and to trade and abides by laws and regulations.	When Nepal wanted to introduce a number of tobacco control measures, the tobacco industry argued that these would adversely 'affect legal tobacco business'[58].
	**F-GA2**. Industry is key economic actor	Industry is engine of economic growth and future prosperity.	Opposing public health policies in Mexico, Coca-Cola reported generating 1.4% of the national GDP and more than 98,000 direct jobs, along with more than a million indirect ones, through the operation of its 67 bottling plants, 350 distribution centers, and 54 wastewater treatment plants[51].
	**F-GA3.** Industry is part of the social fabric	Industry is socially embedded in country/region and part of its history.	In Ireland, opposing a proposed sugar tax, the drinks company Britvic stated: 'The business that we call Britvic Ireland today dates back to 1773 and the invention of Soda Water by Augustine Thwaites Junior in the medical laboratory in Trinity College Dublin' and Lucozade asserted: "Uniquely the food and drink industry is dispersed throughout all regions of Ireland and is at the heart of the social fabric of rural Ireland'[64].
	**F-GA3.** Industry is legitimate policy actor	Industry understands the need to tackle health issues, is reasonable and willing to enter into partnership with government.	In a policy consultation submission, a food and drink company stated: 'our members have expressed their shared commitment to working with the federal agencies to advance the important public health goal of reducing sodium intake'[35].
		Industry has expertise and information that government needs in making policy.	In Thailand, a food company stated: 'collaboration between government and the private sector was the most effective way since the industry is closer to consumers than government'[45].
		Industry needs access to policy spaces and decision-makers because it is part of the solution.	In Canada, a large food company stated in a letter: '… we want to contribute our knowledge and resources to help the government develop and promote a modernized, impactful Canada Food'[59].
	**F-GA4**. Industry is legitimate scientific actor	Industry supports evidence-based policy.	In Australia, the drinks company Red Bull stated: 'Red Bull has long believed and remains firm in its view that sound policy and regulation must be underpinned by evidence-based research'[60].
		Industry is legitimate scientific actor and has expertise in the science of product health harms and solutions.	In the US, the food company Nestle described itself as a 'leader in early childhood nutrition' and added, 'research informs everything (we) do… from the products we make, the nutrition education we deliver and the services we offer'[50].
		Industry is valuable educational resource to the public health community.	In France, CERIN [Centre for Research & Information on Nutrition] advertised itself as 'a research and nutrition information centre and the health department of the

			dairy industry' aiming to 'deliver comprehensive and validated nutritional information about milk and dairy products, but also about the general themes of nutrition and health, nutritional needs of subgroups of the population, and the prevention of pathologies through nutrition, to health and public health professionals, but also to journalists'[43].
	**F-GA5**. Industry is champion of public health	Industry is responsible, committed to prevention of NCDs[1] and working to reduce health harms.	In South Africa, the South African Sugar Association claimed: 'SASA]is concerned about the increase in obesity and NCDs in South Africa. The Association has a longstanding commitment to promoting healthy lifestyles and the prevention of NCDs. This has been demonstrated, for more than 30 years, by its investment in the health of society, especially in rural areas through the support of outdoor gyms, physical activity programmes, wellness events and nutrition education to health professionals and educators'[57].
		Industry supports the proposed policy.	In Canada, an email from a food company to Health Canada (public health agency) stated: 'We support the efforts of Health Canada in this area as we are also very committed to providing easy to understand information to help consumers make healthier food choices'[59].
	**F-GAI6**. Industry is socially responsible	Industry creates welfare by investing in social and economic development and is concerned with social justice.	In Colombia, the food company Alqueria explained that its distribution of products to food banks was crucial for the country: 'We are aware of the importance of our role in the food chain and of our commitment to eradicate hunger in Colombia'[55].
		Industry is committed and essential to sustainable development.	In Canada, a food company stated in a brochure: 'By sourcing responsibly, protecting our environment and making a positive difference in our communities we strive to be an exemplary corporate citizen and use our scale to tackle some of society's toughest challenges'[59].
	**F-GA7.** Industry is victim	Industry is unfairly demonised.	In the US, the alcohol company Miller Brewing argued that a product (beer) which forms a significant part of the local history should not be 'demonised'[7].
**The 'bad' actors: proponents of whole-population, statutory policies**	**F-QA1.** Policymakers who support unfavourable policies have questionable skills and motives	Policymakers may have good intentions but are incompetent/misguided, offering policies that contradict existing policies, are ineffective, illegal or not in keeping with international norms and standards.	In Canada, a food trade association wrote, following a meeting with Health Canada, the regulatory agency, that the latter had 'lost its way on the obesity issue'[59].
		Policymakers are disingenuous, for example, they want to raise revenue, not protect the public's health, or have a hidden agenda, for example to introduce restrictions on other products or industries (*slippery slope*).	In Finland, the alcoholic and soft drinks trade body claimed that the government wanted to introduce a comprehensive alcohol control law because it had a hidden agenda to maintain the state-owned alcohol monopoly, Alko[69].
		Policymakers are authoritarian and want to control people's lives (*nanny-state*).	In opposing the Australian government's alcohol control policies, the alcohol industry described the government as a 'nanny state' needlessly interfering with people's choices'[7].

	**F-QA2.** Public health community have questionable skills and motives	Scientists are incompetent or untrustworthy, engaging in bad scientific practices and promoting false or misleading findings.	In South Africa, opposing a proposed sugary drinks tax, the consumer goods trade association said: "Much of the research reported is either inconclusive, one-sided or from international sources. Further research should follow all the protocols of correct, objective scientific research which requires logic also in its indication of cause and effect. This seems to be lacking in the arguments raised to justify the tax'[57].
		Scientists and advocates are ideologically motivated and have anti-industry/anti-free-enterprise agenda.	Coca Cola labelled those who criticised its sponsorship of the International Congresses of Physical Activity and Public Health 'miscreants' and 'bastards'[56].
		Scientists and advocates are fanatical and want to control the lives of the reasonable/responsible majority.	The alcohol industry often characterizes policymakers and public health actors as authoritarian: 'the health lobby's approach is to ban everything, and if it cannot be banned, regulate it severely'[7].
**<u>The trivial problem</u>: created by a minority of consumers**	**F-P1**. Health harms are not caused by Industry's products/services	Industry's products/ingredients/services are harmless or cause minimal problems.	In Ireland, the publicans' trade association claimed that 'SSBs sold via the licensed trade make a negligible contribution to obesity among children and young people'[64].
		Industry's products/ingredients have been misclassified/confused with other, genuinely harmful products/ingredients.	In South Africa, the South African Fruit Juice Association and Pioneer Foods, whose primary beverage product are 100% fruit juices, did not oppose the adoption of an sugary drinks tax, but opposed the inclusion of 100% fruit juices as taxed beverages[57].
		Health problems have complex causes that cannot be traced to industry products or services alone.	In South Africa, a sugar trade association opposed sugar tax by stating: 'Cane growers is extremely concerned about the increasing trend in obesity and NCDs in South Africa but is of the view that the targeting of an individual ingredient in a particular food product as the tax aims to do, is highly unlikely to resolve a complex health condition that requires a multi-disciplinary approach, including an improvement of the current government health care system'[57].
		Industry's products/services contribute to health, wellbeing and enjoyment of life.	In South Africa, a food and drink trade association stated: 'The hungry and starving in our communities are many and they need affordable energy to be able to live, survive and grow. Sugar provides such a source of affordable energy'[57].
		Industry's products/services are aligned with cultural norms and practices and are used responsibly by the majority.	Opposing alcohol control policies in Portugal, the alcohol industry said: 'In Southern Europe… there is a wine-making tradition, with daily consumptions that accompany meals and in general, a repudiation of drunkenness in public… Southern countries must unite, because when measures are applied there will not be a distinction between wine and vodka'[48].
	**F-P2**. Health harms arise from	Health harms result from individuals' or sub-populations' wrong or uninformed choices and irresponsible behaviours.	In Thailand, an industry sources stated: 'I think if consumers have knowledge, they can make their own healthy food choices. If they know that product is unhealthy or harmful to their health, they shouldn't choose it'[45].

	consumption patterns of atypical minorities	Health harms result from cognitive problems or physical/mental health problems.	In Australia, Clubs Australia, the clubs' trade association, described problem gambling as a 'complex mental health issue influenced by a number of psychological, biological and social factors' and referred to 'faulty cognitions'[16].
	**F-P3**. Health harms are exaggerated	Health harms only affect a minority and are exaggerated by the public health community.	In Australia, the gambling industry argued that: there was no evidence that inducements led to problem gambling; most people were recreational non-problem gamblers; betting was secondary to consumers' interests in sporting events; online betting was not risky; the impact of gambling messages on children was unknown; only a small proportion of the sports viewing audience were children and those who did, watched television with their parents[16].
		There are far more serious and urgent health problems that government should prioritise instead.	We did not have specific examples of this in our dataset. But see the following publications for more information: Muggli ME, Lee K, Gan Q, Ebbert JO, Hurt RD. "Efforts to Reprioritise the Agenda" in China: British American Tobacco's Efforts to Influence Public Policy on Second hand Smoke in China. *PLoS Med*. 2008;5(12):1729-1769. doi:10.1371/journal.pmed.0050251 and Fooks GJ, Gilmore AB (2013) Corporate Philanthropy, Political Influence, and Health Policy. PLoS ONE 8(11): e80864. https://doi.org/10.1371/journal.pone.0080864.
**<u>The acceptable, 'good' solution</u>: individual-focused, industry supported**	**F-S1**. Solutions should target individuals, not whole populations	Solution is to help individuals or 'problem' sub-populations to change their consumption behaviours through information, health education and promotion.	In the US, as food trade association stated: 'in conclusion, the [industry] is responsible and is dedicated to providing meaningful choices and useful information for consumers when making purchasing decisions'[35].
		Solution is to 'treat' problematic consumption using targeted interventions and 'harm reduction' approaches.	In Australia, the gambling industry 'promoted alternative regulatory policy by supporting national voluntary standards for harm minimisation and consumer protection, but on the proviso, they are "achievable from an operational and technical perspective"'[16].
	**F-S2**. Solutions should be self-regulatory & not disrupt business	Self-regulation and voluntary action by industry (on advertising, marketing, labelling, etc) are more effective and more compatible with business operations.	Opposing alcohol control policies in Australia, one company stated: 'marketing self-regulation has advantages over legislation and government regulation. It can address problems that arise quickly and creatively—far more efficiently than a long-drawn-out legal process and it is cost-free to the tax-payer'[60].
**<u>The unacceptable, 'bad' solution</u>:**	**F-NS1**. Policies are unnecessary & unacceptable	Policy is unnecessary because industry is successfully self-regulating and initiating public health interventions.	In Canada, a food company wrote in a letter: 'our members responded positively to the voluntary initiative to reduce/eliminate trans-fat as demonstrated through research and development… Similarly [our] members have made sodium reduction a priority and reduced sodium in pantry bread by 11%'[59].
		Existing regulation is sufficient and should be better enforced before new measures are introduced.	In the UK, the alcohol industry argued: 'the panoply of powers available to the police and local authorities should be used much more effectively both against individuals who misuse alcohol and those who wilfully seek to break the law in

**whole population, statutory**			obtaining alcohol underage, as well as against those retailers who sell alcohol irresponsibly'[7].
		Policy is disproportionate to the problem.	In the UK, the alcohol industry argued that marketing restrictions were 'disproportionate and more extensive than necessary'[7].
		Policy is out of line with global standards and other countries' policies.	In Colombia, the food industry argued that proposed labelling policies were out of line with the Codex Alimentarius[53].
		Policy is regressive and discriminatory.	In Mexico, the soft drinks trade association argued that a proposed sugar tax would 'affect the poorest segment of the population and that it would not achieve a decrease in the consumption of SSB, nor improve health'[51].
		Policy is not evidence-based.	In Australia, an alcohol company stated: 'Despite considerable research on the subject, there is insufficient evidence to support a relationship between advertising and either levels of drinking or patterns of drinking'[60].
	**F-NS2**. Policies/policy formulation contravene norms, rules & laws	Government has not sufficiently consulted industry or other groups.	In South Africa, Coca Cola argued: '[the] proposal on a sugary drinks tax has not received the required scrutiny by key stakeholders… much work still needs to be done for ordinary consumers, informal traders and the lowest income groups to be thoroughly consulted on the implications of the tax for them' (Karim).
		Government has failed to conduct comprehensive social and economic impact assessment.	In South Africa, food and drink industry sources 'requested' that the Treasury be 'required to carry out a comprehensive SEIAS [Socio-Economic Impact Assessment System], with proper engagement with stakeholders'[57].
		The body proposing regulation has no legal authority to do so.	In Brazil, opposing an additives ban, the tobacco industry trade body questioned (in court) the competence of the country's health regulation agency ANVISA to regulate the tobacco industry[47].
		Policy is unconstitutional, impedes basic rights (e.g. the right to free speech) and curtails basic freedoms of a legal business.	In Nepal, the tobacco industry claimed that the public places' smoking legislation violated smokers' rights and the proposed tobacco control law 'prohibited the public's fundamental freedom to conduct trade and business'[58].
		Policy is illegal (e.g. it violates terms of international trade and investment agreements).	In Brazil, opposing a food labelling proposal, the food industry trade association stated in its consultation submission: 'unilateral measures by the Brazilian government may even trigger consultations within the framework of the World Trade Organization for potential violation of the Agreement on Technical Barriers to Trade'[66].
	**F-NS3**. Policies will lead to losses for businesses,	Policy will be impossible to implement (cost)effectively.	In the US, in response to a reformulation consultation, a food company stated: '… our members have not identified available technology that will allow for a significant reduction in sodium without flavor loss, dramatic cost increase, or adding additional questionable ingredients, both from a consumer and restaurant perspective'[35].

	economy & society	Policy implementation will increase administrative cost to governments.	In South Africa, the sugar industry claimed that a proposed sugar tax would impose 'a high administrative burden on revenue collection'[57].
		Policy will reduce competitiveness, innovation and investment and lead to business closures and job losses (especially among SMEs and also in associated sectors like farming).	In Nepal, after a tobacco control law was passed, the tobacco industry claimed that the law's implementation would lead to the loss of 600,000 jobs and closures[58].
		Negative impacts on business will affect the wider economy, reducing GDP[2].	In Canada, in opposing healthy eating strategy legislation, food associations stated that restrictions on marketing to children would lead to '$7 billion in lost GDP, 30,000 plus lost jobs and… more than $300 million in reduced spend with Canada's struggling broadcast industry'[59].
		Policy will discourage foreign investment in the country.	In Nepal, the tobacco industry claimed that the proposed tobacco control laws would 'negate foreign investment and invite instability'[58].
		In LMICs, policy will impede economic development and make LMICs[3] less competitive.	In South Africa, the sugar industry claimed that a proposed sugar tax would curtail 'our ability to enhance our contribution to broader economic growth, job creation and sustainability' and that 'emerging black producers' would have to 'close shop'[57].
		Industry will not be able to support or invest in social justice projects.	Following the suppression of a subsidy on sugar-sweetened beverages, Coca-Cola said that it lost revenues and had to cut 177 jobs, and as a consequence, stopped sponsoring the Colombian soccer team[55].
	**F-NS4**. Policy will fail & have perverse consequences	Policy will not work or has not worked elsewhere.	In Brazil, opposing a proposed food labelling policy, food industry sources stated: 'Two recent surveys released in Chile show that the alarmist nutrition labelling model adopted in Chile does not educate or stimulate change in consumer habits' and '… this type of labelling did not help the consumer as expected'[66].
		Policy is a blunt or simplistic instrument and will not achieve nuanced change.	In Australia, the alcohol industry argued: 'The proposed restrictions on trading hours will not help to stop alcohol-fuelled violence; in fact, it might make it worse'[60(p715)].
		Policy will cause confusion or fear.	In Canada, a food association argued that new nutritional labels on cheese and flavoured yogurt with warning labels could discourage their consumption altogether, despite their scientifically proven nutritional benefits; meanwhile, because potato chips and diet soda would not have such labels, people would perceive these as more healthy'[59].
		Policy will increase illicit trade and smuggling or encourage cross-border shopping.	In Ireland, opposing a proposed sugar tax, Coca-Cola claimed that the policy would create 'the potential for black-market or counterfeit products entering the marketplace which may be mistaken for legitimate soft drink products and not in compliance with food and drink safety standards'[64].
